# A rare *IL33* loss-of-function mutation reduces blood eosinophil counts and protects from asthma

**DOI:** 10.1371/journal.pgen.1006659

**Published:** 2017-03-08

**Authors:** Dirk Smith, Hannes Helgason, Patrick Sulem, Unnur Steina Bjornsdottir, Ai Ching Lim, Gardar Sveinbjornsson, Haruki Hasegawa, Michael Brown, Randal R. Ketchem, Monica Gavala, Logan Garrett, Adalbjorg Jonasdottir, Aslaug Jonasdottir, Asgeir Sigurdsson, Olafur T. Magnusson, Gudmundur I. Eyjolfsson, Isleifur Olafsson, Pall Torfi Onundarson, Olof Sigurdardottir, David Gislason, Thorarinn Gislason, Bjorn Runar Ludviksson, Dora Ludviksdottir, H. Marike Boezen, Andrea Heinzmann, Marcus Krueger, Celeste Porsbjerg, Tarunveer S. Ahluwalia, Johannes Waage, Vibeke Backer, Klaus A. Deichmann, Gerard H. Koppelman, Klaus Bønnelykke, Hans Bisgaard, Gisli Masson, Unnur Thorsteinsdottir, Daniel F. Gudbjartsson, James A. Johnston, Ingileif Jonsdottir, Kari Stefansson

**Affiliations:** 1 Amgen Inc., Discovery Research, South San Francisco, California, United States of America; 2 deCODE genetics / Amgen Inc., Reykjavík, Iceland; 3 School of Engineering and Natural Sciences, University of Iceland, Reykjavik, Iceland; 4 Department of Medicine, Landspitali, The National University Hospital of Iceland, Reykjavik, Iceland; 5 The Laboratory in Mjodd, RAM, Reykjavik, Iceland; 6 Department of Clinical Biochemistry, Landspitali, The National University Hospital of Iceland, Reykjavik, Iceland; 7 Laboratory Hematology, Landspitali, The National University Hospital of Iceland, Reykjavik, Iceland; 8 Faculty of Medicine, School of Health Sciences, University of Iceland, Reykjavik, Iceland; 9 Department of Clinical Biochemistry, Akureyri Hospital, Akureyri, Iceland; 10 Department of Respiratory Medicine and Sleep, Landspitali, The National University Hospital of Iceland, Reykjavik, Iceland; 11 Department of Immunology, Landspitali, The National University Hospital of Iceland, Reykjavik, Iceland; 12 GRIAC research institute, Groningen, The Netherlands; 13 University Medical Center Groningen, University of Groningen, Department of Epidemiology, Groningen, The Netherlands; 14 Center for Pediatrics, Department of General Pediatrics, Adolescent Medicine and Neonatology, Medical Center – University of Freiburg, Faculty of Medicine, Freiburg, Germany; 15 Department of Respiratory Medicine, Bispebjerg University Hospital, Copenhagen University, Copenhagen, Denmark; 16 COPSAC (Copenhagen Prospective Studies on Asthma in Childhood), Herlev and Gentofte Hospital, University of Copenhagen, Copenhagen, Denmark; 17 University Medical Center Groningen, University of Groningen, Department of Pediatric Pulmonology and Pediatric Allergology, Beatrix Children's Hospital, Groningen, The Netherlands; New York Genome Center & Columbia University, UNITED STATES

## Abstract

IL-33 is a tissue-derived cytokine that induces and amplifies eosinophilic inflammation and has emerged as a promising new drug target for asthma and allergic disease. Common variants at *IL33* and *IL1RL1*, encoding the IL-33 receptor ST2, associate with eosinophil counts and asthma. Through whole-genome sequencing and imputation into the Icelandic population, we found a rare variant in *IL33* (NM_001199640:exon7:c.487-1G>C (rs146597587-C), allele frequency = 0.65%) that disrupts a canonical splice acceptor site before the last coding exon. It is also found at low frequency in European populations. rs146597587-C associates with lower eosinophil counts (β = -0.21 SD, *P* = 2.5×10^–16^, N = 103,104), and reduced risk of asthma in Europeans (OR = 0.47; 95%CI: 0.32, 0.70, *P* = 1.8×10^–4^, N cases = 6,465, N controls = 302,977). Heterozygotes have about 40% lower total *IL33* mRNA expression than non-carriers and allele-specific analysis based on RNA sequencing and phased genotypes shows that only 20% of the total expression is from the mutated chromosome. In half of those transcripts the mutation causes retention of the last intron, predicted to result in a premature stop codon that leads to truncation of 66 amino acids. The truncated IL-33 has normal intracellular localization but neither binds IL-33R/ST2 nor activates ST2-expressing cells. Together these data demonstrate that rs146597587-C is a loss of function mutation and support the hypothesis that IL-33 haploinsufficiency protects against asthma.

## Author summary

Only a few genes have been found to play a role in asthma. These include the genes *IL33* and *IL1RL1*, and sequence variants in the human genome close to these genes were initially found to affect the number of eosinophils, cells that play a role in inflammation of the airways in asthma. Based on this knowledge, we decided to use high resolution sequencing technology to search for variants in these genes that cause changes in structure and function of the proteins they encode. We found a rare (0.65%) sequence variant in the *IL33* gene, that causes less production of the IL33 protein and some of the protein formed lacks the capacity to bind to its receptor on cells and promote inflammation. This rare mutation causes reduced number of eosinophils in blood and protects against asthma. These results suggest that drugs that could interfere with the inflammatory activity of the IL33 protein may be beneficial for treatment of asthma.

## Introduction

Asthma is characterized by airflow obstruction, airway hyper-responsiveness and airway inflammation, that promotes mucus obstruction. The inflammatory cytokine IL-33 is widely expressed[1, 2] and abundantly in the bronchial epithelium. IL-33 resides in the nucleus due to chromatin-binding motifs[3] but is released after exposure to e.g. viruses or allergens. IL-33 binds to its receptor ST2[4] (also called IL1RL1, IL-33R) and activates eosinophils and other immune cells promoting inflammation[5], particularly in the lung[6, 7]. IL-33 also binds to a soluble isofrom IL1RL1-a/sST2, which is thought to act as a decoy receptor and ameliorates airway inflammation[8].

Through GWAS, we previously discovered common sequence variants in *IL1RL1* and *IL33* that associate strongly with blood eosinophil counts and risk of asthma, consistent with the link between eosinophilic inflammation and asthma [9]. The association between *IL1RL1* and *IL33* variants and asthma risk is well established [10] and has been replicated in ethnically diverse populations[11, 12], and in severe forms of adult asthma[11] and in particular with early childhood[13] asthma with exacerbations.

The critical role of eosinophilic airway inflammation in asthma[14] together with robust association of eosinophil counts and asthma with common variants at the *IL33* and *IL1RL1* loci prompted us to search for novel sequence variants at these loci affecting eosinophil counts using high-coverage sequencing [15]. The variants were identified through sequencing of 8,453 Icelanders and imputed into 150,656 chip-typed Icelanders[15]. In light of the role of eosinophils in pathogenesis of asthma and the previously established association of common variants at the *IL33* and *IL1RL1* loci with eosinophil counts and asthma, individual sequence variants at these loci found to associate significantly with eosinophil counts were further assessed for their effects on asthma.

## Results

We focus on the association of sequence variants in two 800kb regions centered on *IL33* (chr9:5.8–6.6Mb(hg38)) and *IL1RL1* (chr2:101.9–102.7Mb(hg38)) with eosinophil counts in 103,104 Icelanders[16]. Sequence variants were weighted according to their prior probability of affecting gene function by applying different thresholds for genome-wide (gw) significance that depend on the variant class[17].

**Fig 1:**
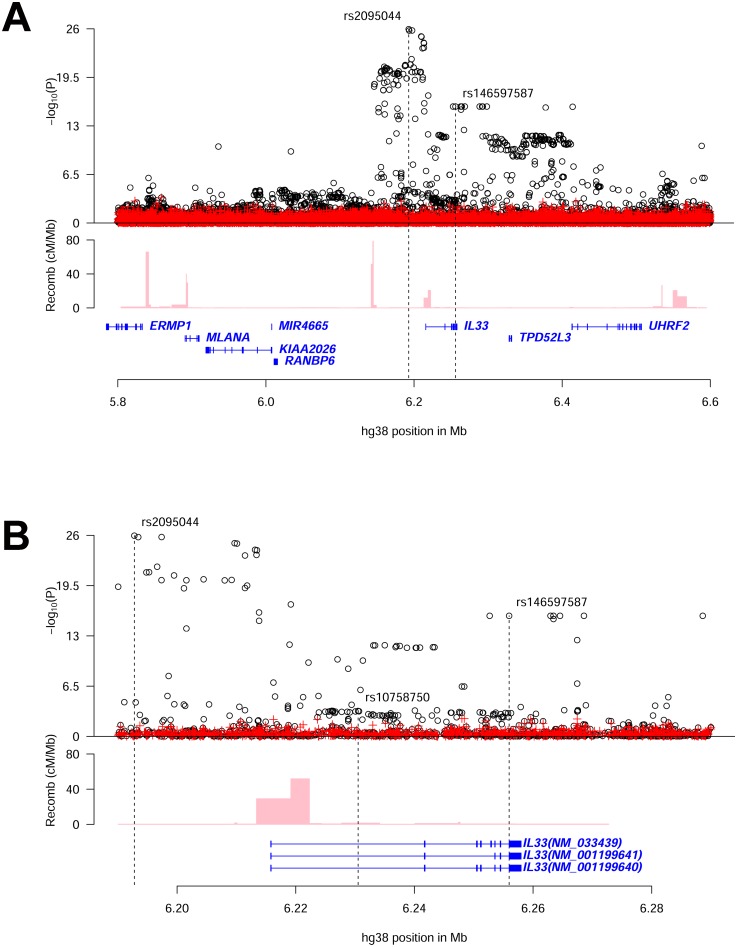
Overview of eosinophil counts associations in the region around *IL33*. **(A)** shows an 800kb overview centered on *IL33* on chromosome 9 and (**B)** shows a 100kb overview around the *IL33* gene. Black circles show -log_10_ *P* as a function of hg38 coordinates for associations with eosinophil counts and red crosses correspond to eosinophil counts associations after adjusting for the three variants rs2095044, rs146597587 (splice acceptor) and rs10758750 that are indicated by vertical broken lines in (**B)**. Genes are shown in blue and recombination rates are reported in cM/Mb. (See S1 Fig for intermediate results from stepwise regression.)

Stepwise conditional analysis at the *IL33* locus revealed two significant uncorrelated (pairwise r^2^<0.02) variants (Fig 1 and S1 Fig, Table 1 and S1 Table). One association is novel: rs146597587-C a rare variant (allelic frequency (AF) = 0.65%) that is predicted to disrupt a canonical splice acceptor site at the beginning of the last exon of *IL33*[18, 19] (NM_001199640:exon7:c.487-1G>C, β_adj_ = -0.21 SD; *P*_adj_ = 2.5×10^–16^) (Table 2). rs146597587-C associates with lower eosinophil counts and is not correlated with previously reported variants at the *IL33* locus (S2 Table). The second variant, rs2095044-T (AF = 24.5%), upstream of *IL33*, is associated with increased eosinophil count (β_adj_ = 0.051 SD; *P*_adj_ = 3.6×10^–27^) and is highly correlated with rs2381416 (r^2^ = 0.94), originally described to associate with eosinophil counts and asthma[9] (Table 1, S3 and S4 Tables). A potential secondary signal was noted, rs10758750-G (AF = 27.7%), an intronic variant in *IL33* (β_adj_ = -0.023 SD; *P*_adj_ = 5.7×10^–7^) (Table 1) (passing a significance threshold of 2.8×10^–6^, corresponding to a Bonferroni correction for the number of variants tested (17,935) at the two loci[20]), uncorrelated (pairwise r^2^<0.02) to the novel variant reported here (Fig 1 and S1 Fig, Table 1 and S1 Table) and to the previously reported variants at the *IL33* locus (S2 Table).

**Table 1:** Association of common sequence variants in *IL33* and *IL1RL1* with eosinophil counts and asthma in Iceland.

Marker	Pos. (hg38)	A1	Freq. A1^b^ [%]	gene context	Eosinophil counts (N = 103,104)				Asthma^a^ (N_affected_ = 3,512, N_controls_ = 298,026)			
					unadjusted		adjusted		unadjusted		adjusted	
					Β^c^ (95%CI)	*P*	β^c^ (95%CI)	*P*	OR^d^ (95%CI)	*P*	OR^d^ (95%CI)	*P*
***IL33* locus:**												
rs2095044	chr9: 6,192,796	T	24.5	intergenic	0.051 (0.04,0.06)	1.1×10^–26^	0.051 (0.04,0.06)	3.6×10^–27^	1.12 (1.04,1.20)	0.0018	1.10 (1.03,1.18)	0.0048
rs10758750^e^	chr9: 6,230,513	G	27.7	Intronic (*IL33*)	-0.016 (-0.03,-0.01)	5.1×10^–4^	-0.023 (-0.03,-0.01)	5.7×10^–7^	1.06 (0.98,1.14)	0.12	1.04 (0.97,1.12)	0.27
***IL1RL1* locus:**												
rs13020553	chr2: 102,315,366	G	41.9	intronic (*IL1RL1*)	0.048 (0.04,0.06)	3.5×10^–31^	0.043 (0.03,0.05)	1.6×10^–24^	1.06 (0.99,1.13)	0.078	1.04 (0.98,1.10)	0.19
rs6719123	chr2: 102,259,080	G	14.2	intergenic	-0.048 (-0.06,-0.04)	1.3×10^–16^	-0.037 (-0.05,-0.03)	7.0×10^–10^	0.92 (0.85,1.00)	0.049	0.92 (0.85,1.01)	0.083

Association results for each variant is presented with and without adjusting for the other variants at its locus in this table. All the variants were well imputed (imputation information = 1.00).

^a)^ The asthma sample set includes young adults, 45 years old or younger[9] (Materials and methods).

^b)^ Freq. A1: Allelic frequency for allele A1.

^c)^ β: Effect in SD with respect to allele A1.

^d)^ OR: Odds ratio with respect to allele A1.

^e)^ rs10758750 shows suggestive association with eosinophil counts, passing a significance threshold of 2.8×10^–6^, corresponding to a Bonferroni correction for the number of variants tested (17,935) at the two loci[20].

**Table 2:** Associations of the *IL33* splice acceptor variant rs146597587[C] with eosinophil counts and asthma in Iceland and abroad.

**Eosinophil counts**	**AF**	**β (SD)**	**(95%CI)**	***P***	**N individuals**		***P***_**het**_**, I**^**2**^
Iceland	0.65%	-0.21	(-0.27, -0.16)	2.5×10^–16^	103,104		
The Netherlands	0.69%	-0.48	(-0.93, -0.03)	0.036	1,370		
**Combined**		-0.22	(-0.27, -0.17)	5.3×10^–17^	104,474		0.25, 25.0
**Asthma**	**AF**	**OR**	**(95%CI)**	***P***	**N cases**	**N controls**	
Iceland:	0.65%	0.36	(0.21, 0.61)	1.2×10^–4^	3,512	298,026	
The Netherlands	0.53%	1.08	(0.36, 3.21)	0.89	351	2,830	
Germany	0.40%	0.89	(0.14, 5.48)	0.90	284	252	
Denmark-1	0.50%	0.72	(0.29, 1.79)	0.48	1,121	1,004	
Denmark-2 (COPSAC)	0.45%	0.24	(0.06, 0.94)	0.04	1,197	865	
**Combined**		0.47	(0.32, 0.70)	1.8×10^–4^	6,465	302,977	0.24, 26.8

Allele frequency (AF) of rs146597587[C], the effect (β (SD)) on eosinophil counts and odds ratio (OR) for asthma and the corresponding *P*-values are provided, in addition to the number of individuals, or cases and controls tested. All the asthma sample sets include children and/or young adults: Iceland 45 years age or younger[9], The Netherlands younger than 45 years of age[23, 42], Germany 5–18 years of age[24], Denmark-1 14 to 44 years of age[25, 26] and Denmark-2 (COPSAC) children with severe asthma with at least 2 exacerbations leading to hospitalization between 2 and 6 years of age[13] (Materials and methods).

Among the two significant variants, the splice acceptor mutation rs146597587-C has the largest effect. The splice acceptor mutation is present in both Europeans (AF = 0.35%) and South-Asians (AF = 0.10%) in the Exome Aggregation Consortium (ExAC) database where it is >10 times more frequent than any other predicted loss-of-function variant in *IL33* (S5 Table, **URLs)**. In total we tested six predicted missense, splice region or loss-of-function variants in *IL33* (S6 Table) and only the association with the splice acceptor mutation rs146597587-C is significant (*P*>0.2 for the others). None of the twelve variants highly correlated (r^2^>0.8) with the splice acceptor mutation were coding, making it most likely to be responsible for the effect (S7 Table). Neither of the two other associating variants, rs2095044 nor rs10758750, were correlated with a coding variant (S4 and S8 Tables).

The *IL33* splice acceptor mutation, rs146597587-C was genotyped in 1,370 Dutch samples and its effect on eosinophil counts was replicated (β = -0.48 SD, *P* = 0.036, AF = 0.69%) (Table 2).

Once the eosinophil count association of the splice acceptor variant was established, we assessed its effect on asthma, based on the prior association of IL33 and with asthma risk and the role of eosinophils in the pathogenesis of asthma. We assessed the effect of rs146597587-C on asthma in Iceland[9], The Netherlands[21–23], Germany [24] and Denmark[25, 26], including Danish children with severe asthma with at least 2 exacerbations leading to hospitalization between 2 and 6 years of age (COPSAC_exacerbation_)[13]. The *IL33* splice acceptor mutation protects against asthma (OR = 0.47; 95% CI: 0.32–0.70, *P* = 1.8×10^–4^) (Table 2). We did not observe heterogeneity of the effect in the different sample sets (*P*_het_ = 0.24, I^2^ = 26.8). Thus, this rare splice acceptor mutation reduces eosinophil counts and protects against asthma, whereas the minor allele of the common rs2095044-T associates with higher eosinophil counts and increased risk of asthma (Tables 1 and 2).

**Fig 2:**
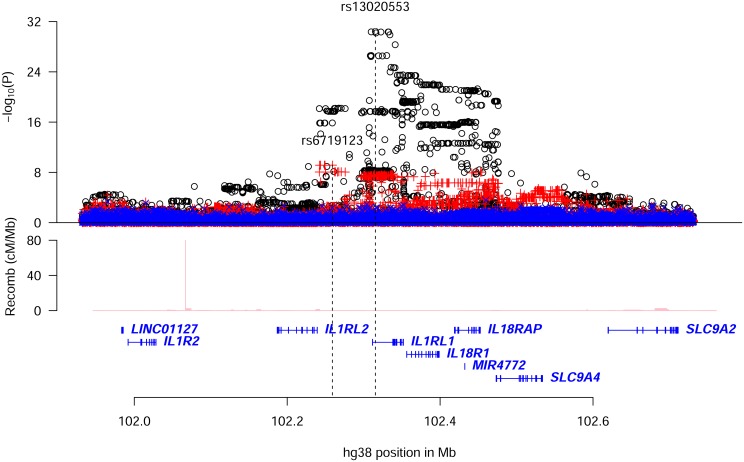
Conditional analysis for eosinophil counts associations in the region around *IL1RL1*. Plot shows an 800kb overview around the *IL1RL1* gene on chromosome 2. Black circles (o) show-log_10_ *P* as a function of hg38 coordinates for unadjusted associations with eosinophil counts; red crosses (+) correspond to eosinophil counts associations after adjusting for the variant rs13020553; blue ‘x’ symbols correspond to eosinophil counts associations after adjusting for both rs13020553 and rs6719123. The position of the two variants rs13020553 and rs6719123 are indicated by vertical broken lines. Genes are shown in blue and recombination rates are reported in cM/Mb.

Stepwise conditional analysis of the association with eosinophil counts (N = 103,104) at the *IL1RL1* locus revealed two significant variants, an intronic variant, rs13020553-G (MAF = 41.9%), increasing eosinophil counts (β = 0.043, P_adj_ = 1.6×10^–24^) and an intergenic variant, rs6719123-G (MAF = 14.2%), decreasing eosinophil counts (β = -0.037, P_adj_ = 7.0×10^–10^) (Table 1 and S9 Table); no other variant at the locus remained significant after adjusting for these two (Fig 2 and S10 Table). rs13020553 is highly correlated with rs1420101 (r^2^ = 0.96, D’ = 1.00), originally reported to associate with eosinophil counts and asthma[9] (S3 and S10 Tables). Despite the high correlation, rs1420101-T (that is on the background of rs13020553-G) does not fully explain the eosinophil association of rs13020553, whereas rs13020553 fully explains the eosinophil association for rs1420101 (S10 Table). Together rs13020553 and rs6719123 explain all the reported effects on eosinophil counts and asthma at the locus (S3 Table); however, the reported variants do not fully explain the signal captured by rs13020553 and rs6719123 (S11 Table).

We found and tested 75 coding or splice region variants at the *IL1RL1* locus. Among those not in *IL1RL1*, only one was significant (*P*>0.001 for all other coding variants not in *IL1RL1*), rs1420098-C, a splice region variant in *IL18R1* (MAF = 42.5%, *P* = 3.8×10^–24^, β = 0.042). The eosinophil counts association of rs1420098 was fully accounted for by rs13020553 (r^2^ = 0.81, P_adj_ = 0.53), whereas the association of rs13020553 could not be accounted for by rs1420098 (P_adj_ = 1.2×10^–8^, β_adj_ = 0.054). We observed two significant coding signals in *IL1RL1* among the sixteen detected and tested (14 missense, one splice region variant and one frameshift variant) (S12 Table). Both coding signals were previously reported to associate with concentration of ST2 in soluble form (sST2)[27]. The first, represented by rs10192157-T (MAF = 39.0%, *P* = 4.2×10^–20^; HGVSp: NP_057316.3:p.Thr549Ile), corresponds to five perfectly correlated missense variants (r^2^ = 1.00, D’ = 1.00 for all pairs) moderately correlated with rs13020553 (r^2^ = 0.35); the second rs1041973-A (MAF = 17.7%, *P* = 3.5×10^–9^, HGVSp: NP_057316.3:p.Ala78Glu) is correlated with rs6719123 (r^2^ = 0.68) (S13 Table). These two eosinophil counts associations are fully accounted for by rs13020553 and rs6719123 (S14 Table). We conclude that the eosinophil counts signals corresponding to rs13020553 and rs6719123 (correlated variants: S15 and S16 Tables) could not be explained by the observed coding variants or reported variants at the locus. Our results are in agreement with the notion that variants at this locus influence eosinophil counts by affecting *IL1RL1*; and IL-33 is known to mediate its biological effects through ST2/IL1RL1[4].

**Fig 3:**
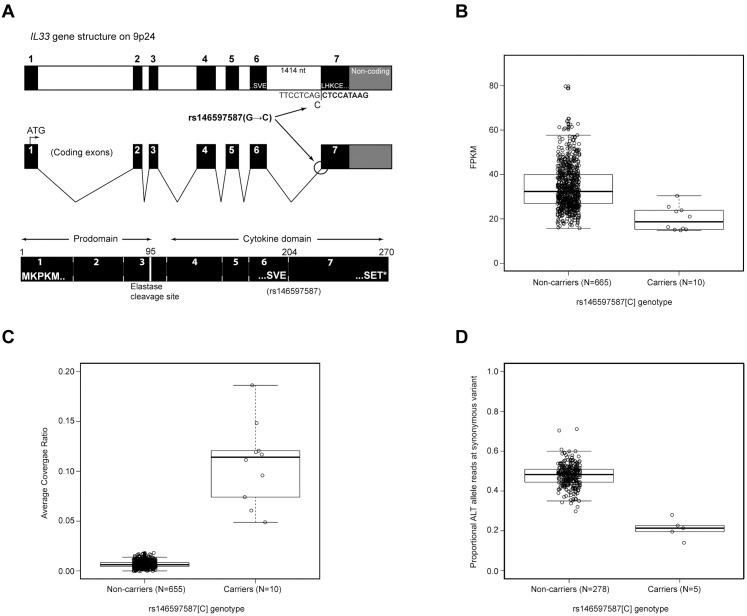
RNA analysis of *IL33* mRNA expression and carrier status of the splice acceptor variant rs146597587. **(A)** Gene structure of *IL33* and location of rs146597587 SNP that changes a canonical splice acceptor site from AG to AC. The mutation lies at the junction of coding exons 6 and 7. Exon 7 begins LHKCE and encodes a significant portion of the cytokine domain. Also, indicated is the location of an elastase cleavage site at amino acid 95 that releases the cytokine domain from the N terminus prodomain and leads to a more active cytokine. **(B)** Expression in FPKM of *IL33* in adipose tissue for rs146597587 non-carriers (GG, N = 744) and carriers (GC, N = 6) based on RNA-Seq data; *P(Wilcox)* = 0.0015, median non-carriers: 34.1, carriers: 21.1 (i.e. heterozygotes have 38% lower expression than non-carriers). **(C)** Ratio of RNA-Seq read coverage in last intron of *IL33* versus its last exon (splice acceptor resides at this intron-exon boundary) for rs146597587 non-carriers (GG, N = 738) and carriers (GC, N = 9); *P*(Wilcox) = 2.5×10^–7^, mean non-carriers: 0.67%, carriers: 9.0%. **(D)** Proportion of RNA-Seq read with ALT allele for the synonymous variant rs10975519 (MAF = 29%) for rs146597587 non-carriers (GG, N = 309) and carriers (GC, N = 5); a chromosome carrying rs146597587-C carries the ALT allele of rs10975519; *P*(Wilcox) = 1.4×10^–4^, mean non-carriers: 0.48, non-carriers: 0.20.

The splice acceptor mutation in *IL33* changes AG to AC at the 3’ splice junction between the last two exons and is thus predicted to disrupt splicing between them [18, 19]. *IL33* is primarily expressed by stromal cells and is expressed at a relatively low level in hematopoetic tissues. Of the two tissues with mRNA sequencing data available to us *IL33* is essentially absent from whole blood but is expressed in subcutaneous adipose tissue[1] (median RPKM of 12.9 in 350 samples), according to the GTExV6 database[2]. We therefore analyzed the effect of the splice acceptor mutation on the *IL33* transcript quantity and processing using the adipose tissue mRNA sequencing (N = 675) dataset (Materials and methods**, and** Fig 3). Heterozygote carriers (N = 10) of the splice acceptor mutation showed about 40% lower total *IL33* expression than non-carriers (*P* = 6.8×10^–6^) (Fig 3). Comparable results were obtained by accessing total IL-33 expression with microarray (S2 Fig). Allele-specific analysis of the RNA sequencing data shows that only 20% of total *IL33* transcripts are from the mutated chromosomes (*P* = 1.3×10^–4^) indicating that a large fraction of mRNA originating from the mutated chromosomes is likely eliminated through nonsense-mediated decay (NMD)[28]. Analysis of read coverage in the RNA sequencing data, shows retention of the last intron of *IL33* in about half of the RNA generated from the mutated chromosome (~11% of total RNA) in heterozygotes compared to 0.6% of that in non-carriers (*P* = 5.6×10^–8^). Taken together these data demonstrate that the splice acceptor mutation leads both to elimination of the mutated transcripts and a retention of the last intron, introducing a premature stop codon in about half of the remaining mutated transcripts.

**Fig 4:**
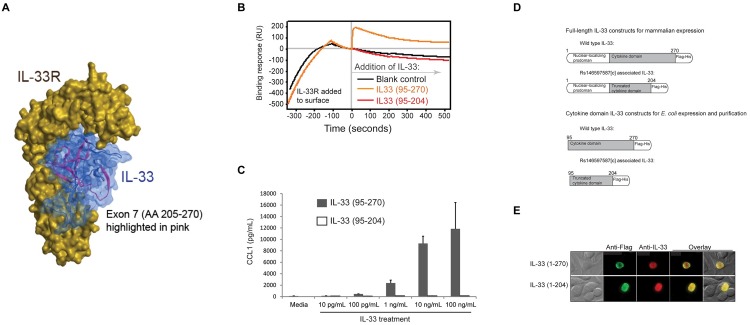
(**A**) Computational model of the IL-33 cytokine domain bound to its receptor. Shown in pink are amino acids 205–270, which are derived from exon 7 and absent in the rs146597587-C-associated truncated form of IL-33. Besides contributing to overall structure, this part of IL-33 also contributes to receptor interaction. (**B**) Binding profile from Biacore surface plasmon resonance measurement of IL-33 binding to IL-33R. Recombinant IL-33R encompassing the extracellular domain was immobilized on the chip surface and then recombinant IL-33 was allowed to flow over the chip (beginning at time 0). Productive receptor binding is indicated by a prolonged increase in resonance following cytokine addition. Wild type IL-33 (95–270) bound receptor, but the mutant IL-33 (95–204) did not. (**C**) Biological response to IL-33. LAD2 human mast cells were treated with the indicated concentrations of either wild type IL-33 (95–207) or mutant IL-33 (95–204) and concentration of CCL1 released into the culture medium was measured by ELISA. (**D**) Schemata of constructs made to evaluate expression of full-length IL-33 proteins in mammalian cells or, for expression and purification of cytokine domain-only proteins in *E*. *coli*. (**E**) Immunofluorescent staining indicating equivalent nuclear localization of full-length wild type IL-33 (1–270) and the rs146597587[C]-associated form of IL-33 (1–204) in transfected HEK293 human epithelial cells.

The IL-33 protein generated from the intronic retention transcripts is predicted to lack the last 66 amino acids (out of 270). IL-33 folds as a 12-stranded “β-barrel” structure, prototypical for the IL-1 family. Elimination of the last 66 amino acids removes 5 of the 12 core β-strands and is predicted to disrupt the tertiary structure of the protein. To determine whether IL-33 lacking these residues is functional we expressed recombinant IL-33 lacking the last exon (termination at residue 204, Fig 4). IL-33_1–204_ was detectable in transfected mammalian cellular lysates as a 27kD protein, compared to the 35 kD wild type IL-33_1–270_ (S3 Fig). IL-33_1–204_ was in the nucleus in a manner indistinguishable from IL-33_1–270_ (Fig 4), indicating that the truncation does not disrupt nuclear trafficking.

Computational modeling [29] indicates that truncated IL-33 lacks core structural folds and key surface residues predicted to contribute to receptor binding (Fig 4A). To determine whether the truncated form binds ST2 we generated IL-33_95–270_ (wild type) and IL-33_95–204_ (truncated mutant) recombinant proteins. The N-terminus starting residue at amino acid position 95 was chosen based on natural processing of IL-33 before receptor binding[30] (S4 Fig).

**Fig 5:**
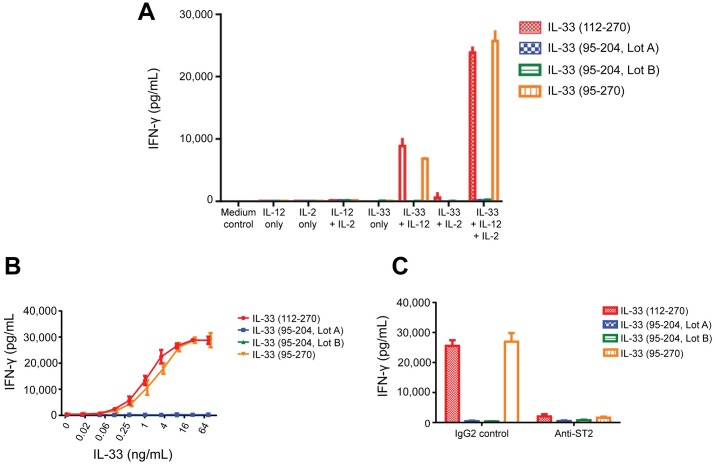
Activity of IL-33 variants in human CD4 T cell bioassay. Purified human blood CD4 T cells were incubated in the presence or absence of IL-2 and IL-12 for 72 hours and IFN-γ was measured in the cell-free supernatants (as described in online methods). Both IL-33 (95–270) (Amgen) and IL-33(112–270) (R&D Systems) were used as positive controls and two independent lots of IL-33(95–204) were tested. (**A**) IL-33, IL-2 and IL-12 dependence of assay with IL-33 at 10ng/mL; (**B**) Dose titration of IL-33 variants (in the presence of 10 ng/mL IL-2 and 10 ng/mL IL-12). (**C**) ST2 dependence of response. IL-33 variants (10 ng/mL plus IL-2 and IL-12) were tested in the presence of 25 ug/mL human IgG2 control antibody or IgG2 anti-human ST2 blocking antibody.

Using surface plasmon resonance we found that IL-33_95–270_ bound rapidly to IL-33R/ST2 (Fig 4), consistent with high affinity binding to IL-33R that we previously reported[31], whereas IL-33_95–204_ showed no interaction signal, indicating a complete lack of receptor binding. Furthermore, IL-33_95–270_ induced a concentration- and IL-33R-dependent CCL1 release in human mast cell line expressing IL-33R (Fig 4C and S5 Fig), whereas IL-33_95–204_ did not at any concentration tested, consistent with a complete loss of cytokine activity. The mutant IL-33 was also inactive compared to wild type inducing IFN-γ release from human CD4^+^ T cells (Fig 5).

**Fig 6:**
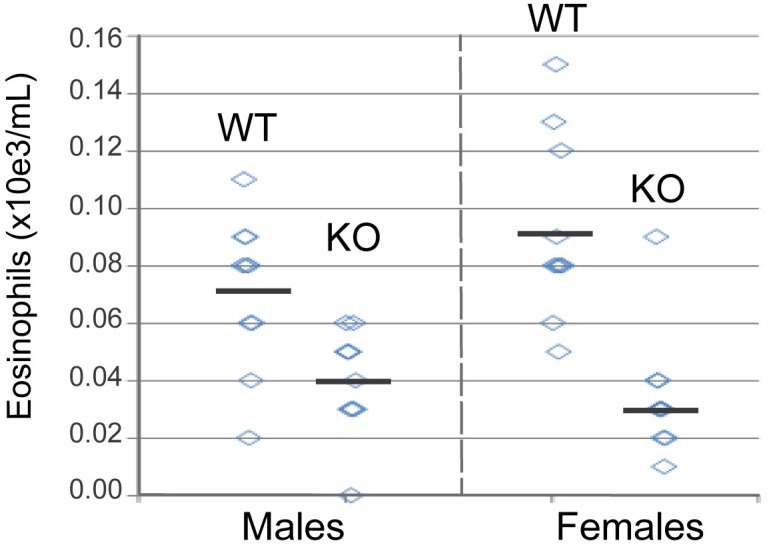
Blood eosinophil counts from wild type and *il33*-deficient mice. Blood were collected from male and female 10–12 week old mice and eosinophils were enumerated as described in the online methods. The horizontal bars indicate means within groups. Significance calculated using the unpaired T test, p = 0.014 males, p = 0.0001 females.

In *il33* knock-out mice, 30% reduced fertility by has been reported[32]. Nine imputed rs146597587-C homozygotes were found in the Icelandic data and they neither show reduced life expectancy nor reduced fertility (S16 Table), indicating that a predicted very low level of IL-33 is compatible with long life and healthy reproduction. Moreover, rs146597587-C homozygosity (recessive model) does not confer risk of any other disease that was tested in the Icelandic data[15, 33], indicating that IL-33 is largely dispensable. Two homozygotes of the splice acceptor mutation are reported in ExAC (S5 Table, **URLs**). An association of *il33* polymorphism with eosinophil numbers in rats has been reported[34] and we found that deletion of *il33* leads to significant reduction in blood eosinophils in unchallenged mice (*P* = 0.014 males, *P* = 0.00010 females) (Fig 6), mimicking our human observations.

## Discussion

Resequencing of 100 genes implicated in asthma only revealed a few coding variants associating with asthma[35]. Since eosinophils are known to play a key role in inflammation of the airway in asthma[12] we used high-coverage sequencing [13] to search for novel sequence variants affecting eosinophil counts at the well established asthma loci, the *IL33* and *IL1RL1* loci, and tested their effects on asthma. Our results indicate that variants at the *IL1RL1* locus affect eosinophil counts and the risk of asthma most likely by affecting *IL1RL1* itself. IL-33 is known to mediate its biological effects through ST2/IL1RL1[4] and sensitized mice with *il1rl1* (ST2-/-) knocked out show less eosinophil numbers in bronchoalveolar lavage fluid upon allergen exposure than wild-type mice, reduced levels of Th2 cytokines and chemoattractants in the lungs, and reduced goblet cell hyperplasia around the peripheral airways in murine models of allergy and asthma[36, 37].

We report a rare variant, rs146597587, in *IL33* representing a loss-of-function mutation in this known asthma gene. This splice acceptor mutation associates with lower eosinophil counts and protection against asthma, with the largest protective effect from severe asthma with frequent exacerbations in young Danish children (OR = 0.24, Table 2). This is in agreement with increasing risk conferred by the common *IL33* variant rs2381416 with increasing severity of asthma in these children, with OR from 1.27 to 1.69 for severity groups 1 to 4 (S17 Table). The splice acceptor mutation caused reduced expression of *IL33* transcripts, likely due to NMD, and production of truncated IL-33 that lacks the cytokine function due to lack of binding to IL-33R/ST2 resulting in abrogation of IL-33R-dependent release of CCL1 from mast cells and IFN-γ release from human CD4^+^ T cells. We therefore infer that asthma risk is mediated through IL-33. Accordingly, common variants in *IL1RL1* that associate with increased asthma risk associate with reduced expression of soluble ST2[38, 39], with the predicted effect being increased IL-33 activity, due to reduced level of this decoy receptor. Thus, human genetics support the rationale for therapeutically inhibiting the IL-33-ST2 pathway in an attempt at containing asthma.

## Materials and methods

### Ethics statement

The Icelandic study was approved by the National Bioethics Committee (VSN_14–099) and the Data Protection Authority (no. PV_2014060841/ÞS) in Iceland. All participating subjects who donated blood provided informed consent. Personal identities of the participants and biological samples were encrypted by a third-party system approved and monitored by the Icelandic Data Protection Authority.

The Denmark-1 study was approved by the local ethics committee of Copenhagen, Denmark (Approval no. KF01-400/98 and KF01-074/01). All participants signed informed consent.

The COPSAC study research protocol was approved by The Danish National Ethical Committee on Health Research (KF 01-289/96, H-B-2008-093, H-16039498 and H-B-2998-103) and is in accordance with the ethical scientific principles of the Helsinki Declaration II. All parents in the cohorts signed informed consent.

The German study was approved by the Ethical Commission of the University of Freiburg (Approval no. 96/05). All participants signed informed consent.

The Dutch European ancestry asthma study was approved by the Medical Ethics Committee of the University Hospital Groningen (Approvals no. MEC 90/09/178, MEC 96/04/077, MEC 97/10/184). All participants signed informed consent. The Dutch Vlagtwedde/Vlaardingen study protocol was approved by the local university medical hospital ethics committee, University of Groningen, University Medical Center Groningen, The Netherlands and all participants gave their written informed consent. In 1984, the Committee on Human Subjects in Research of the University of Groningen reviewed the study and affirmed the safety of the protocol and study design.

The mouse experiments were performed at Amgen Inc. All animal use procedures were in accordance with Amgen animal use and care guidelines and approved by IACUC.

### Study sample sets for eosinophil count and asthma

#### Iceland

We obtained eosinophil counts from three of the largest laboratories in Iceland (measurements performed between the years 1993 and 2015). The median eosinophil counts were 200/μl (lower and upper quartiles: 100/μl and 300/μl). The circulating eosinophil counts were standardized to a standard normal distribution using quantile-quantile standardization and then adjusted for sex, year of birth and age at measurement, as previously described[9, 40]. A total of 103,104 individuals with eosinophil counts were included in the study, where 82,642 were chip-typed and directly imputed; the remaining 20,462 were first and second degree relatives of chip-typed individuals and had their genotypes inferred based on genealogy[16].

Icelandic asthma patients over 18 years of age who attended an asthma clinic or emergency room at the National University Hospital of Iceland or the Icelandic Medical Center (Laeknasetrid) during the years 1977 to 2014 were recruited[9]. Asthma diagnosis was based on a combination of physician’s diagnosis, a positive reply to the question: „Has a doctor confirmed your asthma diagnosis?“, questionnaires pertaining to asthma symptoms and ICD diagnosis when receiving emergency care[9, 40]. Atopy status (defined by at least one positive response to allergens) determined by skin prick testing was available for part of the cohort. A total of 3,512 (1,842 chip-typed) asthma cases 45 years old or younger were used in the study (18.5% with known age of onset 18 years of age or younger) [9, 40] and 298,026 (134,762 chip-typed) controls. Icelandic controls were participants from various deCODE genetics programs without known asthma.

The study was approved by the National Bioethics Committee (VSN_14–099) and the Data Protection Authority (no. PV_2014060841/ÞS) in Iceland. All participating subjects who donated blood provided informed consent. Personal identities of the participants and biological samples were encrypted by a third-party system approved and monitored by the Icelandic Data Protection Authority.

#### Denmark 1

Asthma subjects and controls from three studies at the Copenhagen Research Unit, aged 14 to 44 years and resided in Copenhagen, Denmark, were used in the analysis[25, 26]. The asthma patients had doctor diagnosed asthma by a respiratory specialist and had undergone a physical examination and spirometry. Danish control subjects were recruited through the Danish Blood Donor Corps in the Copenhagen area.

#### Denmark 2 (COPSAC)

Cases were from the COPSAC_exacerbation_ cohort. Children with repeated acute hospitalizations (cases) were identified in the Danish National Patient Register covering all diagnoses of discharges from Danish hospitals[41]. Information on birth-related events was obtained from the national birth register. Inclusion criteria were at least two acute hospitalizations for asthma (ICD-8-code 493, ICD-10 codes J45-46) from 2 to 6 years of age, as previously described[13]. Duration of hospitalization had to be more than 1 day, and two hospitalizations had to be separated by at least 6 months. Exclusion criteria were side diagnosis during hospitalization, registered chronic diagnosis considered to affect risk of hospitalization for asthma, low birth weight (<2.5 kg) or gestational age of under 36 weeks at birth. Control subjects were children without asthma exacerbations in the COPSAC_2000_ and COPSAC_2010_ birth cohorts. Study participants gave informed consent for use of their biological samples for genetic studies. The research protocol was approved by The Danish National Ethical Committee on Health Research and is in accordance with the ethical scientific principles of the Helsinki Declaration II.

#### Germany

The German case-control population consisted of children (aged 5–18 years) with suspected asthma recruited from the southwestern part of Germany between July 2000 and January 2005[24]. The probands were characterized at the Centre of Pediatrics and Adolescent Medicine, Freiburg, Germany. The diagnosis of asthma was based on a history of respiratory symptoms, the use of anti-asthmatic medication and the presence of bronchial hyperresponsiveness (defined as a fall of at least 15% in baseline FEV1 after either inhalation of ≤8 mg/ml histamine or ≤6 minutes of exercise provocation). Participants were asked in advance to discontinue any asthma or allergy medication before the clinical testing. Atopy status was determined by skin prick tests to 17 common allergens. Measurement of total serum IgE was carried out by using an enzyme allergosorbent test (Phadezym; Pharmacia, Uppsala, Sweden). The control sample consisted of subjects (aged 19–40 years) randomly chosen from the same area in the southwestern part of Germany and children recruited from clinics at the Centre of Pediatrics and Adolescent Medicine, Freiburg, Germany. No medical history was taken, and no medical testing was performed on the adult controls whereas the pediatric controls had no previous history of asthma, recurrent wheezing, atopic dermatitis or atopy. Approval was granted by the Ethical Commission of the University of Freiburg. A statement of informed consent was signed by all participants or signed by their parents in the case of children.

#### The Netherlands

The Dutch cases have been previously described[21–23]. Briefly asthma patients of European ancestry were initially studied between 1962 and 1975. At the time of recruitment and initial testing, all patients had asthma symptoms, were hyperresponsive to histamine (PC20 histamine <32 mg/ml), and were younger than 45 years of age. Between 1990 and 1999, patients were invited for follow-up tests: including pulmonary function, bronchial responsiveness to histamine, and total and specific serum IgE and skin tests to 16 common aero allergens [22, 23]. Participants were asked in advance to discontinue asthma or allergy medication, therapy with oral corticosteroids was however continued.

A second independent Dutch asthmatic population was ascertained between 1998 and 2001, consisting of probands with asthma, ascertained through local hospitals and media appeals. Controls were derived from Vlagtwedde/Vlaardingen cohort study, a population-based cohort of adult subjects of European ancestry in the Netherlands, recruited from 1965 with a follow-up of over 25 years. Surveys were performed every 3 years, in which information was collected on respiratory symptoms, smoking status, FEV1, and allergy skin tests [42].

### Whole-genome sequencing and imputation

Genotyping of all Icelandic samples was carried out at deCODE genetics in Reykjavik, Iceland, using methods recently described[15]. In brief, whole-genome sequencing was performed for 8,453 Icelanders who were recruited as part of various genetic programs at deCODE genetics, to an average depth of at least 10× (median 32×) using Illumina technology. The sequencing was performed using the following three different library preparation methods and sequencing instruments from Illumina: (i) standard TruSeq DNA library preparation method; Illumina GAIIx and/or HiSeq 2000 sequencers; (ii) TruSeq DNA PCR-free library preparation method; Illumina HiSeq 2500 sequencers; and (iii) TruSeq Nano DNA library preparation method; Illumina HiSeq X sequencers (S1 Method).

SNPs and indels identified in the whole-genome sequencing data were identified using the Genome Analysis Toolkit HaplotypeCaller (GATK version 3.3.0)[43] and imputed into 150,656 Icelanders who had been genotyped with various Illumina SNP chips and their genotypes phased using long-range phasing chip-genotyped individuals using long-range phasing[44]. In addition, using the Icelandic genealogical database, genotype probabilities were calculated for first- and second-degree relatives of chip-genotyped individuals[15]. The effects of sequence variants on protein-coding RefSeq genes[18] were annotated using the Variant Effect Predictor (VEP) version 80[19].

### Genotyping of single variants

Single SNP genotyping in the replication sample sets was carried out by deCODE Genetics in Reykjavik, Iceland, applying the Centaurus (Nanogen) platform[45] except that genotyping single SNPs in the Danish cohort of children with severe asthma was carried out at the AROS Applied Biotechnology AS center (http://arosab.com/services/microarrays/genotyping/), in Aarhus, Denmark, applying the Illumina Infinium HumanOmniExpressExome Bead chip platform and genome studio software[46].

### Association testing in Iceland

Generalized linear regression models were used to test for associations between sequence variants and quantitative traits, assuming an additive genetic model. Let y be the vector of quantitative measurements, and let g be the vector of expected allele counts for the sequence variant being tested. We assume the quantitative measurements follow a normal distribution with a mean that depends linearly on the expected allele at the variant and a variance covariance matrix proportional to the kinship matrix:

$$y\sim N\left(\alpha +\beta g,\;2\sigma^{2}\Phi \right),$$

where

$$\Phi_{ij}=\{\begin{array}{ll} \frac{1}{2}, & i=j\\ 2k_{ij}, & i\neq j \end{array}.$$

is the kinship matrix as estimated from the Icelandic genealogical database. Logistic regression was used to test for association between sequence variants and binary traits. Other available individual characteristics that correlate with disease status were also included in the model as nuisance variables. These characteristics were: sex, county of birth, current age or age at death (first- and second-order terms included), blood sample availability for the individual and an indicator function for the overlap of the lifetime of the individual with the timespan of phenotype collection. Testing was performed using the likelihood ratio statistic. Conditional analysis was performed by including the sequence variant being conditioned on as a covariate in the model under the null and the alternative in the generalized linear regression. Sequence variants were weighted according to their prior probability of affecting gene function by applying different thresholds for genome-wide (gw) significance that depend on the variant class[17].

Association summary statistics are provided in supplementary tables for the *IL33* locus (S18 Table) and the *IL1RL1* locus (S19 Table).

#### LD score regression

To account for inflation in test statistics due to cryptic relatedness and stratification, we applied the method of LD score regression[47]. With a set of 1.1M variant we regressed the χ^2^ statistics from GWAS scans against LD score and used the intercept as a correction factor. The LD scores were downloaded from an LD score database (see **URLs**). The correction factors were 1.2844 for the eosinophil counts, and 1.1120 for asthma.

### Gene expression microarrays

Samples of RNA from human peripheral blood and adipose tissue were hybridized to Agilent Technologies Human 25K microarrays as described previously[48] and the effect of SNPs on the *IL33* expression in adipocytes evaluated. We quantified expression changes between two samples as the mean logarithm (log10) expression ratio (MLR) compared to a reference pool RNA sample. In comparing expression levels between groups of individuals with different genotypes, we denoted the expression level for each genotype as 10 (average MLR), where the MLR is averaged over individuals with the particular genotype. We determined s.e.m. and significance by regressing the MLR values against the number of risk alleles carried. We took into account the effects of age, gender and differential cell type count in blood as explanatory variables in the regression.

### RNA sequencing

The effect of on the *IL33* expression in adipocytes was evaluated by RNA sequencing as previously described[20].

#### Preparation of Poly-A cDNA sequencing libraries

The quality and quantity of isolated total RNA samples was assessed using the Total RNA 6000 Nano chip for the Agilent 2100 Bioanalyzer. cDNA libraries derived from Poly-A mRNA were generated using Illumina‘s TruSeq RNA Sample Prep Kit. Briefly, Poly-A mRNA was isolated from total RNA samples (1–4 μg input) using hybridizaton to Poly-T beads. The Poly-A mRNA was fragmented at 94°C, and first-strand cDNA was prepared using random hexamers and the SuperScript II reverse transcriptase (Invitrogen). Following second-strand cDNA synthesis, end repair, addition of a single A base, adaptor ligation, AMPure bead purification, and PCR amplification, the resulting cDNA was measured on a Bioanalyzer using the DNA 1000 Lab Chip.

#### Sequencing

Samples were clustered on to flowcells using Illumina‘s cBot and the TruSeq PE cluster kits v2, respectively. Paired-end sequencing was performed with either GAIIx instruments using the TruSeq SBS kits v5 from Illumina or HiSeq 2000 instruments using TruSeq v3 flowcells/SBS kits; read lengths were 2x76, 2x101 or 2x125 cycles.

#### Read alignment

RNA sequencing reads were aligned to Homo sapiens Build 38 with TopHat[49] version 2.0.12 with a supplied set of known transcripts in GTF format (RefSeq hg38; Homo sapiens, NCBI, build 38). TopHat was configured such that it attempts first to align reads to the provided transcriptome then, for reads that do not map fully to the transcriptome, it attempts to map them onto the genome. Read mapping statistics used for read count normalization were calculated using the CollectRnaSeqMetrics tool in Picard version 1.79 (http://broadinstitute.github.io/picard/command-line-overview.html#CollectRnaSeqMetrics). After the read alignment step by TopHat, gene expression estimation in FPKM values (Fragments Per Kilobase of transcript per Million mapped reads) was done using Cufflinks[49, 50] version 2.2.1 for the same set of transcripts used in the read alignment by TopHat.

### Mammalian expression

IL-33 variant-expressing vectors as shown in Fig 4D were transfected into HEK293–EBNA1 cells (obtained from National Research Council of Canada) using the protocols described elsewhere[51]. At 24 hours post transfection, cells were either seeded onto polylysine-coated glass coverslips and cultured for 24 hours for imaging, or fed with Difco yeastolate cell culture supplement (BD Biosciences). Cell culture media were collected and whole cell lysates were prepared 2, 4, and 7 days post transfection.

### Cell imaging

At 48 hrs post transfection, cells were fixed with 4% paraformaldehyde in 0.1 M sodium phosphate, pH 7.2, for 30 minutes at room temperature. After a wash in PBS containing 0.1 M glycine, the fixed cells were incubated with permeabilization buffer (PBS containing 0.4% saponin, 1% BSA, 5% fish gelatin) for 15 minutes, followed by incubation with primary antibody in permeabilization buffer for 60 min. Primary antibodies included anti-IL33 mature domain (R&D Systems), anti-FLAG (clone M2, Amgen), anti-GAPDH (clone 6C5, Amgen), anti-gianin (Covance) and anti-GM130 (BD Transduction Lab). After 3 washes in permeabilization buffer, the cells were incubated with Alexa Fluor 488- or 594-conjugated secondary antibody in permeabilization buffer for 60 minutes. Slides were analyzed using a Nikon Eclipse 80i microscope using a 100× or 60× CFI Plan Apo oil objective lens. Images were acquired using a Cool SNAP HQ2 digital camera (Photometrics) and Nikon Elements imaging software.

### Western blotting

Harvested cell culture media and processed cell pellets were heated for 5 min at 90°C in lithium dodecyl sulfate sample buffer (Life Technologies) containing 5% (v/v) beta-mercaptoethanol. NuPAGE 4–12% Bis–Tris gradient gel and the accompanying running buffer system (both from Life Technologies) were used to perform SDS-PAGE. Resolved proteins were electrotransferred to a nitrocellulose membrane, blocked with fluorescent Western blocking buffer (Rockland), and probed with primary antibodies (described above). After 3–4 washes in PBS containing 0.05% (v/v) Tween-20, the nitrocellulose membranes were incubated with AlexaFluor 680- conjugated secondary antibodies (Life Technologies), followed by 2–3 additional washing steps in PBS containing 0.05% (v/v) Tween-20. The fluorescent Western images were acquired by using an Odyssey infrared imaging system from LI-COR Biosciences.

### *E*. *coli* expression and purification

IL-33 was cloned into the pET24a expression plasmid in frame with an amino terminus poly-His tag and thrombin cleavage site such that thrombin cleavage would release IL-33 proteins corresponding to residues 95–270 (wild type) or 95–204 (mutant).

For IL-33_95–270_, following *E*. *coli* culture, cell pellets were lysed and the supernatants were collected and purified on a nickel HisTrap column (GE Healthcare Life Sciences). The material was eluted from the column using an imidazole gradient. Appropriate fractions were pooled and further purified by preparative size exclusion chromatography (SEC) using a Superdex 75 column (GE Healthcare Life Sciences) with PBS, pH 7.2, 1 mM DTT as the running buffer. Recovered material was cleaved using the Thrombin CleanCleave Kit (Sigma) with a 1 hour incubation at 4°C. Preparative SEC was used to remove the free His tag and any aggregated material, followed by an additional SEC step to further reduce endotoxin contamination. IL-33_95–270_ was formulated in PBS, pH 7.2, 1 mM DTT, 1 mM EDTA and filtered through a 0.2 μm filter. Endotoxin was undetectable.

IL-33_95–204_ was not recoverable from the cell pellet supernatant due to low expression in the soluble fraction. Instead, inclusion bodies were solubilized 1/10 (w/v) with 8 M guanidine, 50 mM Tris, 8mM DTT, pH 9.0 for 1 hour. This was followed by dilution 1/25 (v/v) with PBS buffer, pH 7.6 and the material was concentrated and loaded onto a Superdex 75 column using PBS, pH 7.2 as the running buffer. The huIL-33 peak was then buffer-exchanged into 1X thrombin cleavage buffer (50 mM Tris-HCl, pH 8.0, 100 mM CaCl_2_) and concentrated to 1 mg/mL. 0.1 mL of thrombin agarose resin (Sigma) was added to 1 mg of huIL-33 and rotated at 4°C for 1 hour. Cleaved huIL-33 was then passed over a HisTrap column to capture free cleaved His tag. The flow-through material was further purified using preparative SEC, as for IL-33_95–270_, prior to formulation in PBS, pH 7.2, 1 mM EDTA. Fractions were analyzed by SDS-PAGE and Coomassie staining (total protein) or Western blot (with anti-IL-33) before and after thrombin cleavage to confirm cleavage and IL-33 identity (S4 Fig).

### Computational modeling

The structure model of the ST2/IL-33 complex was provided by Lingel et al.[29]. The receptor and ligand are shown with a molecular surface, computed with default parameters in the Molecular Operating Environment (MOE, Chemical Computing Group Inc., Montreal, QC, Canada, H3A 2R7, 2011. (n.d.)), with ST2 shown in gold and IL-33 shown in blue, with amino acids 205–270, which are derived from exon 7, shown in magenta (Fig 4A).

### Surface plasmon resonance

All experiments were performed using a Biacore T200 optical biosensor (Biacore AB). HBS-P buffer (10 mM HEPES, pH 7.4, 150 mM NaCl, 0.05% Surfactant P20) was purchased from Teknova. The CM5 sensor chip, sodium dodecyl sulphate (SDS) (0.5% w/v), NaOH (50 mM), coupling reagents (N-ethyl-N’-(3-dimethylaminopropyl)-carbodiimide hydrochloride (EDC)/ N-hydroxy-succinimide(NHS)), ethanolamine (1.0 M Ethanolamine-HCl, pH 8.5), Acetate 5.0 (10 mM sodium acetate) and Glycine 1.5 (10 mM glycine, pH 1.5) were purchased from GE Healthcare Life Sciences. The HCl solution (100 mM) was purchased from Bio Rad Labs. The penta-His antibody (BSA-free) was purchased from 5Prime. All other reagents were prepared by Amgen.

Biosensor analysis was conducted at 25°C in HBS-P buffer. The CM5 sensor chip was conditioned with twice with serial injections (30 seconds) of SDS (0.1%), NaOH (10 mM), HCl (10 mM) and again with SDS (0.1%) at a flow rate of 30 uL/min (first two injections) or 60 ul/min (last three injections). The penta-His antibody was diluted (200 ug/mL) in water and then buffer exchanged via desalting spin column (Zeba, 0.5 mL, 40 K) into Acetate 5.0 buffer. This buffer-exchanged antibody was immobilized to flow cells 1 (3148 RU) and 2 (7554 RU) of the sensor chip via standard amine coupling (EDC/NHS) and ethanolamine blocking[52]. Human ST2-Flag-His (8 ug/mL in HBS-P, Amgen) was injected (300s at 10 uL/min) over flow cell 2. This captured between 600 and 900 RU of antibody. After the capture step, the human *E*. *coli*-derived IL-33 variants (200 nM) were injected (180s, 50 ul/min) over flow cells 1–2 to observe the association (180 s) of human IL-33 to human ST2. Each flow cell was then flushed with running buffer to observe the dissociation of human IL-33 (300 s) from the chip surface. Each sample of IL-33 was tested individually as a single replicate. A blank buffer injection was tested before the first sample injection and the surface was regenerated (15 ul at 50 uL/min) with 10 mM glycine pH 1.5 and a reloading of ST2 was captured to the chip surface before each sample injection.

The data was prepared and analyzed with Scrubber 2.0 software (BioLogic Software Pty Ltd, Campbell, Australia) as follows. The raw data from each experiment was x and y-axis normalized just prior to the injection of IL-33 and then cropped to include the ST2 capture step and the association/dissociation of IL-33.

### Mast cell bioassay

LAD2 human mast cells were seeded into 48-well tissue culture plates at 250,000 cells/375 μL media (Stem-Pro-34 serum-free media (Life Technologies, Grand Island, NY, USA) supplemented with 2 mM L-glutamine, 100 IU/ml penicillin, 50 μg/ml streptomycin (complete SFM) and 100ng/ml SCF (Peprotech). The following day cells were stimulated with medium only or increasing concentrations (10 pg/mL– 100 ng/mL) of wild-type (95–270) or mutant (95–204) IL-33 for 24 hours at 37°C. Culture supernatants were then collected followed by removal of cell debris by centrifugation and stored at -80°C until ready for use. The concentration CCL1 in the collected supernatants was determined using an R&D Human CCL1 DuoSet ELISA kit under manufacturer’s specifications.

To demonstrate the ST2-dependence of the assay, stimulations were performed as described with the addition of a final concentration of 20 ug/mL human IgG2 isotype control (Amgen) or human anti-huST2 blocking antibody (Amgen) 30 minutes prior to addition of 100 ng/mL IL-33.

### Human CD4 T cell bioassay

Highly purified human CD4+ T cells were seeded at 200,000 cells/well in 96-well round bottom plates. E. coli-produced human IL-33, either beginning at amino acid 95, as described above, or using IL-33 (112–270) (R&D Systems) was either titrated as a dose-response or added to a final concentration of 10ng/mL along with human IL-12 and human IL-2, each at a final concentration of 10ng/mL. For ST2 blocking, anti-huST2 IgG2 or IgG2 control was added to a final concentration of 25 ug/mL 30 minutes prior to addition of cytokines. Cultures were incubated for 72hrs at 37°C in a 5% CO2 incubator. Cell-free supernatants were collected and IFN-γ was quantitated by ELISA (R&D Systems).

### Mouse eosinophil enumeration

Blood was collected from 10–12 week old wild-type and il33-deficient Bl/6 under isoflurane anesthesia and analyzed on a Siemens Advia 120 hematology analyzer with mouse-specific software. Blood smears were air-dried and stained with Wright-Giemsa. Automated differentials generated on the Advia 120 were confirmed by slide evaluation for any sample with eosinophil counts greater than 4% or with evidence of platelet clumps on the Advia scatterplots or on the blood smear.

### URLs

Predicted loss-of-function variants in *IL33* and their genotype counts in the Exome Aggregation Consortium (ExAC) database were retrieved from http://exac.broadinstitute.org/gene/ENSG00000137033, accessed November 24, 2015.

LD Score database (accessed 23 June 2015), ftp://atguftp.mgh.harvard.edu/brendan/1k_eur_r2_hm3snps_se_weights.RDS

## Supporting information

S1 Methods Whole-genome sequencing and imputation. Sample preparation and DNA whole-genome sequencing methods:
Sample preparation and sequencing using the standard TruSeq DNA library preparation method.Sample preparation and sequencing using the TruSeq DNA PCR-free method.Sample preparation and sequencing using the TruSeq Nano DNA method.
(DOCX)

S1 Fig Conditional analysis for eosinophil counts associations in the region around IL33. (DOCX)

S2 Fig *IL33* mRNA expression in adipose tissue based on microarray data and carrier status of the splice acceptor variant rs146597587. (DOCX)

S3 Fig Western blot of mammalian cell-expressed human (FLAG-tagged) IL-33 protein variants. (DOCX)

S4 Fig SDS-PAGE analysis of *E*.*coli*-produced IL-33 95–204 variant. (DOCX)

S5 Fig ST2-dependence of human mast cell bioassay. (DOCX)

S1 Table D' and r^2^ between the three variants at *IL33* associating with eosinophil counts. (DOCX)

S2 Table Correlations (r^2^) between reported variants at *IL33* and the three significant eosinophil counts variants. (DOCX)

S3 Table Association results in Iceland for variants at the *IL33* and *IL1RL1* loci reported to associate with asthma, allergy or eosinophil counts in Iceland. (DOCX)

S4 Table Variants that have r^2^>0.8 with the intergenic variant rs2095044 in an 800kb window centered on *IL33* (chr9:5.8–6.6Mb (hg38)). (DOCX)

S5 Table Loss-of-function variants in *IL33* reported in the Exome Aggregation Consortium (ExAC) dataset. (DOCX)

S6 Table Predicted loss-of-function, missense and splice region variants in IL33 that are detected, imputed and tested in Iceland. (DOCX)

S7 Table Variants that have r2>0.8 with the splice acceptor variant rs146597587 in an 800kb window centered on IL33 (chr9:5.8–6.6Mb (hg38)). (DOCX)

S8 Table Variants that have r2>0.8 with the intronic variant rs10758750 in a 800kb window centered on IL33 (chr9:5.8–6.6Mb (hg38)). (DOCX)

S9 Table Correlations (r2) of reported variants at IL1RL1 with the two variants from stepwise regression and two top coding signals. (DOCX)

S10 Table Conditional analysis based on eosinophil counts for top variants from stepwise regression and reported variants at the IL1RL1 locus. (DOCX)

S11 Table Variants that have r^2^>0.8 with the intronic variant rs13020553 in an 800kb window centered on *IL1RL1* (101.9–102.7Mb). (DOCX)

S12 Table Variants that have r2>0.8 with the intergenic variant rs6719123 in an 800kb window centered on IL1RL1 (101.9–102.7Mb). (DOCX)

S13 Table Correlations (r^2^) for the two variants from stepwise regression and two top coding signals at *IL1RL1*. (DOCX)

S14 Table Conditional analysis based on eosinophil counts for top variants from stepwise regression and top coding variants at the *IL1RL1* locus. (DOCX)

S15 Table Predicted loss-of-function, missense and splice region variants in IL1RL1 that are detected, imputed and tested in Iceland. (DOCX)

S16 Table Fertility and longevity data for the nine imputed homozygotes for the splice acceptor variant rs146597587 in IL33 found in Iceland. (DOCX)

S17 Table Association of the common IL-33 variant rs2381416 with pediatric asthma of increasing severity. (DOCX)

S18 Table Associations of sequence variants in a 800kb region centered on IL33 (chr9:5.8–6.6Mb (hg38) with eosinophil counts in 103,104 Icelanders. Shown are results with P<0.05.
(XLSX)

S19 Table Associations of sequence variants in a 800kb region centered on IL1RL1 (chr2:101.9–102.7Mb (hg38) with eosinophil counts in 103,104 Icelanders. Shown are results with P<0.05.
(XLSX)
