# Supplementary material for: A rare IL33 loss-of-function mutation reduces blood eosinophil counts and protects from asthma
Source: PLoS Genet. 2017 Mar 8;13(3):e1006659. doi: 10.1371/journal.pgen.1006659 (PMC5362243; doi:10.1371/journal.pgen.1006659)
Supplement: S1 Methods — Sample preparation and DNA whole-genome sequencing methods: Sample preparation and sequencing using the standard TruSeq DNA library preparation method.Sample preparation and sequencing using the TruSeq DNA PCR-free method.Sample preparation and sequencing using the TruSeq Nano DNA method. (DOCX) [file pgen.1006659.s001.docx]

**Supporting Information**

**S1 Method. Whole-genome sequencing and imputation. Sample preparation and DNA whole-genome sequencing methods.**

Our dataset contains samples obtained using three different library preparation methods from Illumina. In addition sequencing was performed using three different types of Illumina sequencing instruments.

1. Standard TruSeq DNA library preparation method. Illumina GAIIx and/or HiSeq 2000 sequencers*.*
2. TruSeq DNA PCR-free library preparation method. Illumina HiSeq 2500 sequencers.
3. TruSeq Nano DNA library preparation method. Illumina HiSeq X sequencers.

A more detailed description of each sample preparation method is provided below.

***Sample preparation and sequencing using the standard TruSeq DNA library preparation method.*** Approximately 1 μg of genomic DNA, isolated from frozen blood samples, was fragmented to a mean target size of approximately 300-400 bp using a Covaris E210 instrument. The resulting fragmented DNA was end repaired using T4 and Klenow polymerases and T4 polynucleotide kinase with 10 mM dNTP followed by addition of an 'A' base at the ends using Klenow exo fragment (3′ to 5′-exo minus) and dATP (1 mM). Sequencing adaptors containing 'T' overhangs were ligated to the DNA products followed by agarose (2%) gel electrophoresis. Fragments of about 450-500 bp were isolated from the gels (QIAGEN Gel Extraction Kit), and the adaptor-modified DNA fragments were PCR enriched for ten cycles using Phusion DNA polymerase (Finnzymes Oy) and a PCR primer cocktail needed for paired-end sequencing. Enriched libraries were purified using AMPure XP beads. The quality and concentration of the libraries were assessed with the Agilent 2100 Bioanalyzer using the DNA 1000 LabChip. Libraries were stored at −20 °C. Sequencing-by-synthesis (SBS) was performed on either Illumina GAII_x_ or HiSeq 2000 instruments, respectively. Paired-end libraries were sequenced using 2x76, 2x101 or 2x120 cycles of incorporation and imaging with Illumina SBS kits, TruSeq™ v5 for the GAIIx. For the HiSeq 2000, 2x101 cycles with SBS kits v2.5 or v3 were employed. Each library was initially run on a single lane on a GAII_x_ for validation, assessing optimal cluster densities, insert size, duplication rates and comparison to chip genotyping data. Following validation, the desired sequencing depth (10X to 30X) was then obtained using either sequencing platform. Targeted raw cluster densities ranged from 500–800 K/mm^2^, depending on the version of both the sequencing chemistry and the data imaging/analysis software packages (SCS.2.8/RTA1.8 or SCS2.9/RTA1.9 for the GAII_x_ and HCS1.3.8. or HCS1.4.8 for HiSeq 2000). Real-time analysis involved conversion of image data to base-calling in real-time.

***Sample preparation and sequencing using the TruSeq DNA PCR-free method****.* Paired-end libraries for sequencing were prepared according to the manufacturer's instructions (Illumina, TruSeq DNA PCR-free ™). In short, approximately 1 μg of genomic DNA, isolated from frozen blood samples, was fragmented to a mean target size of 350 bp using a Covaris E210  ultrasonicator followed by clean-up using AmPure XP purification beads. Blunt-end DNA from the resulting fragments was generated using a mix of 3’>5’ exonuclease and 5’>3’ polymerase activities, respectively, followed by 5’-phosphorylation using T4 polynucleotide kinase. Size-selection of the blunt-end fragments was done using a two-step purification strategy with different ratios of the AmPure XP purification beads (0.6X and 1X). Finally, 3’-adenylation and ligation of barcoded adapters was performed, followed by clean-up with magnetic beads. The quality and concentration of the libraries were assessed with the Agilent 2100 Bioanalyzer using the DNA 1000 LabChip (Agilent). Barcoded libraries were stored at −20 °C. All steps in the workflow were monitored using an in-house laboratory information management system with barcode tracking of all samples and reagents. All samples were first pooled (12-24 plex) and sequenced on Illumina´s MiSeq instruments (2x25 cycles) to assess quality and effective concentration of sequencing libraries. Subsequent deep sequencing was done on HiSeq 2500 instruments, were each sample was sequenced on 3 lanes, generating >100 Gb of raw data and at least 30X coverage. Sequencing was done using TruSeq v3 reagents, paired-end 2x100 cycles. System operation and base calling in real-time was done using HCS 2.2.38 and RTA 1.18.61.

***Sample preparation and sequencing using the TruSeq Nano DNA method.*** The sample preparation workflow was essentially the same as described above for the TruSeq DNA PCR-free method, except the input amount was 100 ng of genomic DNA (instead of 1 μg) and following clean-up of adapter ligated DNA, the samples were enriched by 8-cycles of PCR using a PCR primer cocktail, followed by Ampure XP bead clean-up. The quality and concentration of the libraries were assessed with the Perkin Elmer LabChip GX instrument using the HT DNA HiSens reagent kit. Sequencing was done using the HiSeq X HD reagent kit. Each sample was loaded onto the HiSeq X instrument at a concentration of 300 pM and sequenced to high depth (>30X). System operation and base calling in real-time was done using HCSX 3.1.26 and RTA2 2.3.9.
