# Supplementary material for: A rare IL33 loss-of-function mutation reduces blood eosinophil counts and protects from asthma
Source: PLoS Genet. 2017 Mar 8;13(3):e1006659. doi: 10.1371/journal.pgen.1006659 (PMC5362243; doi:10.1371/journal.pgen.1006659)
Supplement: S1 Fig — (DOCX) [file pgen.1006659.s002.docx]

**
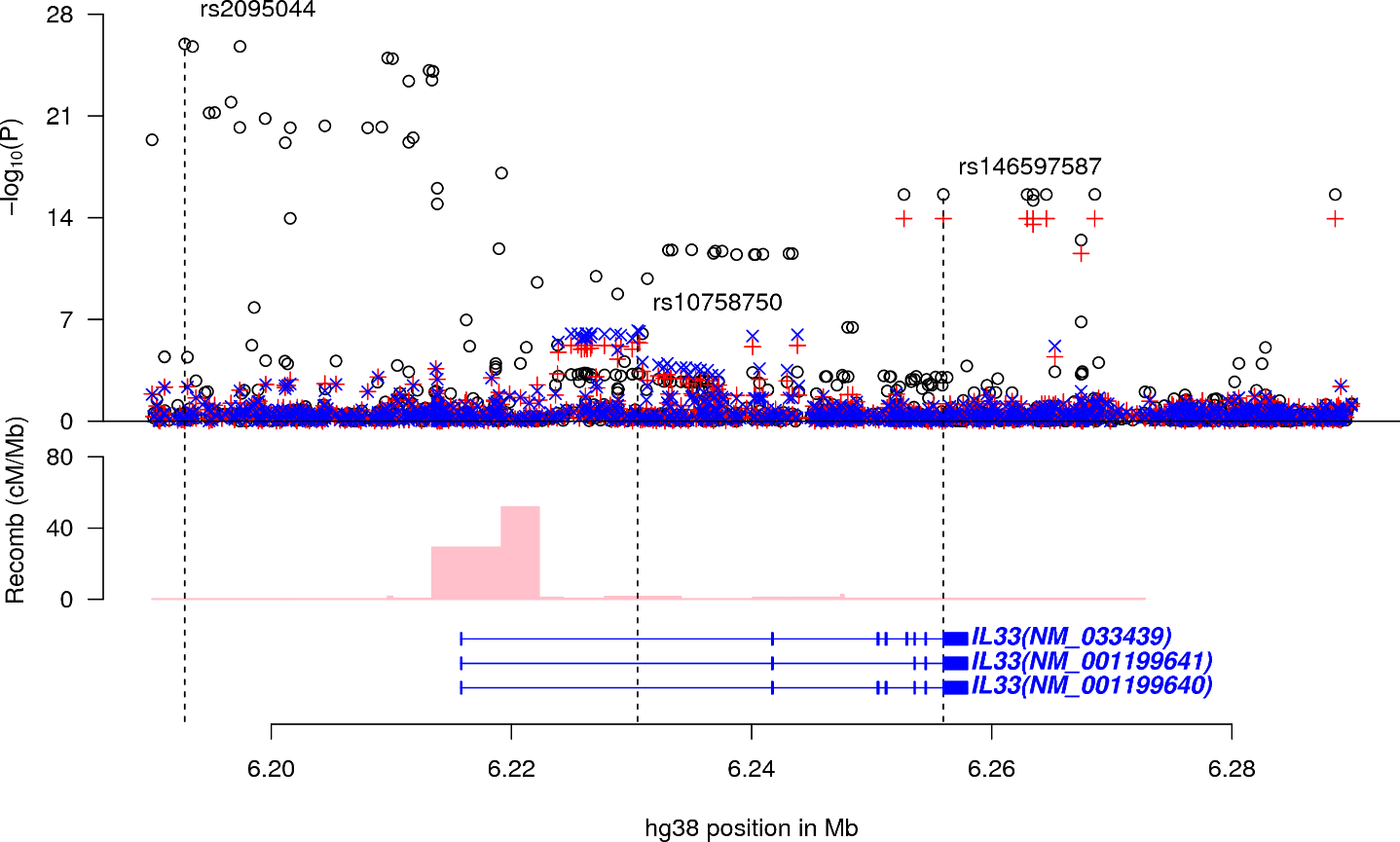
**

**S1 Fig. Conditional analysis for eosinophil counts associations in the region around *IL33*.** Plot is a 100kb overview around the *IL33* gene on chromosome 9. Black circles (o) show-log_10_ *P* as a function of hg38 coordinates for unadjusted associations with eosinophil counts; red crosses (+) correspond to eosinophil counts associations after adjusting for the intergenic variant rs2095044; blue ‘x’ symbols correspond to eosinophil counts associations after adjusting for both rs2095044 and the splice acceptor variant rs146597587. The position of the three variants rs2095044, rs146597587 and rs10758750 are indicated by vertical broken lines. Genes are shown in blue and recombination rates are reported in cM/Mb.
