# Supplementary material for: A rare IL33 loss-of-function mutation reduces blood eosinophil counts and protects from asthma
Source: PLoS Genet. 2017 Mar 8;13(3):e1006659. doi: 10.1371/journal.pgen.1006659 (PMC5362243; doi:10.1371/journal.pgen.1006659)
Supplement: S2 Fig — (DOCX) [file pgen.1006659.s003.docx]

**S2 Fig.
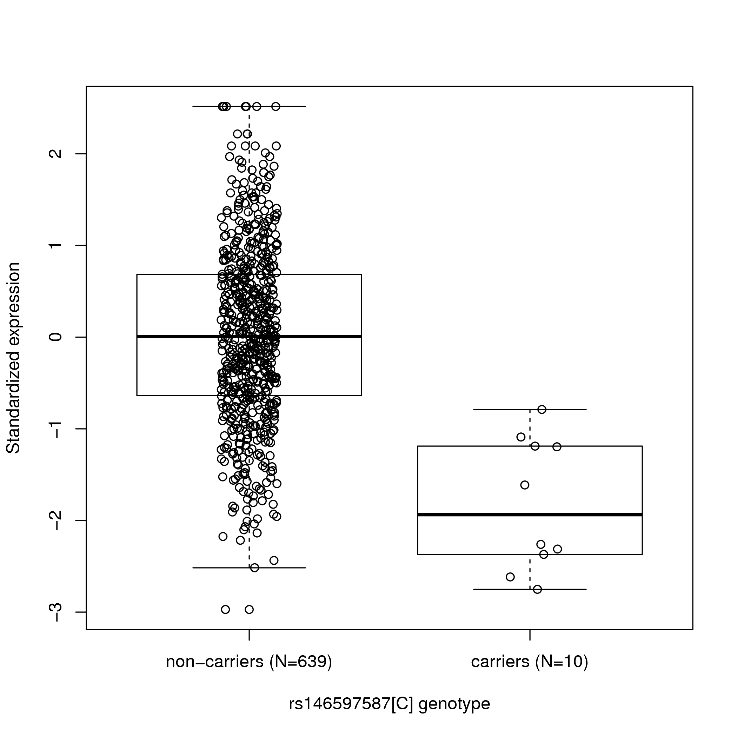
 *IL33* mRNA expression in adipose tissue based on microarray data and carrier status of the splice acceptor variant rs146597587*.*** Expression of *IL33* in adipose tissue for rs146597587 non-carriers (GG, N=639) and carriers (GC, N=10) based on microarray data; P=1.6×10^-7^, β=-1.8 SD, based on regression of the average log expression ratio on the carrier status, adjusting for age and sex, and differential counts for blood.
