# Supplementary material for: A rare IL33 loss-of-function mutation reduces blood eosinophil counts and protects from asthma
Source: PLoS Genet. 2017 Mar 8;13(3):e1006659. doi: 10.1371/journal.pgen.1006659 (PMC5362243; doi:10.1371/journal.pgen.1006659)
Supplement: S3 Fig — (DOCX) [file pgen.1006659.s004.docx]

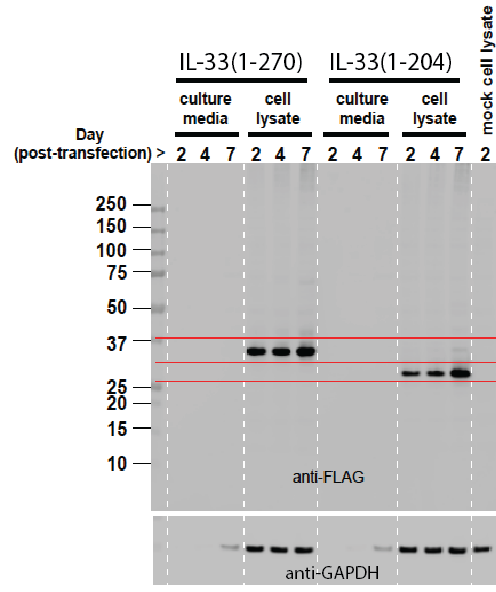


**S3 Fig.** **Western blot of mammalian cell-expressed human (FLAG-tagged) IL-33 protein variants.** Molecular weight markers (in kD) are indicated on left. After SDS-PAGE separation and transfer to nitrocellulose of samples that were collected at day 2, 4 or 7 following transfection, membranes were stained with anti-FLAG antibody to indicate IL-33 and anti-GAPDH to control for protein loading.
