# Supplementary material for: A rare IL33 loss-of-function mutation reduces blood eosinophil counts and protects from asthma
Source: PLoS Genet. 2017 Mar 8;13(3):e1006659. doi: 10.1371/journal.pgen.1006659 (PMC5362243; doi:10.1371/journal.pgen.1006659)
Supplement: S4 Fig — (DOCX) [file pgen.1006659.s005.docx]

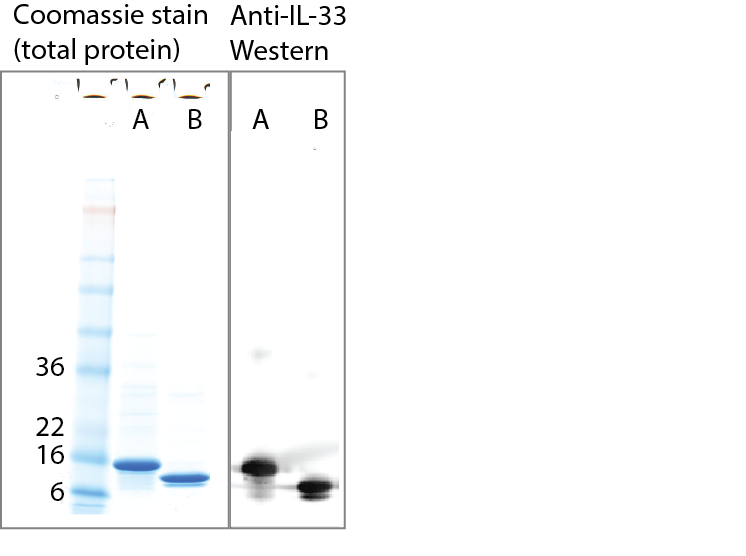


**S4 Fig. SDS-PAGE analysis of *E.coli*-produced IL-33 95-204 variant.** On the left is total protein staining with Coomassie blue and molecular weight markers and sizes (in kD) are indicated. On the right is Western analysis of the same samples using anti-hu IL-33 antibody (R&D Systems). Lanes A: Sample fractions before thrombin cleavage (as described in the online methods); Lanes B: Fractions following thrombin cleavage.
