# Supplementary material for: A rare IL33 loss-of-function mutation reduces blood eosinophil counts and protects from asthma
Source: PLoS Genet. 2017 Mar 8;13(3):e1006659. doi: 10.1371/journal.pgen.1006659 (PMC5362243; doi:10.1371/journal.pgen.1006659)
Supplement: S5 Fig — (DOCX) [file pgen.1006659.s006.docx]

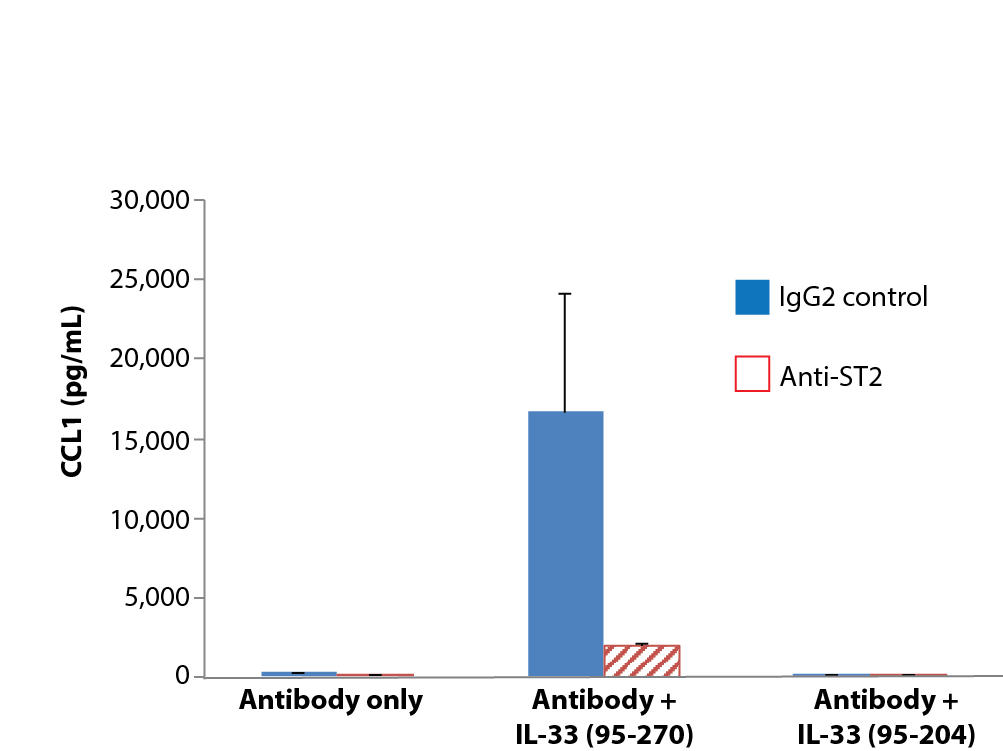


**S5 Fig. ST2-dependence of human mast cell bioassay.** LAD2 mast cells were incubated with IL-33 at 100 ng/mL in the presence of 20 ug/mL human IgG2 control or IgG2 anti-ST2 blocking antibody. CCL1 was measured in the supernatants after 24 hours.
