# Supplementary material for: A rare IL33 loss-of-function mutation reduces blood eosinophil counts and protects from asthma
Source: PLoS Genet. 2017 Mar 8;13(3):e1006659. doi: 10.1371/journal.pgen.1006659 (PMC5362243; doi:10.1371/journal.pgen.1006659)
Supplement: S1 Table — (DOCX) [file pgen.1006659.s007.docx]

**Table S1. D' and r^2^ between the three variants at *IL33* associating with eosinophil counts.**

|  |  |  |  | **Eosinophil counts** | |  | **sign of correlation** |  |
| --- | --- | --- | --- | --- | --- | --- | --- | --- |
| **Variant 1** | **Variant 2** | **f_1_ [%]** | **f_2_ [%]** | **β_1_** | **β_2_** | **r^2^** |  | **D’** |
| rs2095044-T | rs146597587-C | 24.5 | 0.65 | 0.049 | -0.201 | 0.0021 | -1 | 1.00 |
| rs2095044-T | rs10758750-G | 24.5 | 27.7 | 0.053 | -0.021 | 0.012 | 1 | 0.12 |
| rs146597587-C | rs10758750-G | 0.65 | 27.7 | -0.219 | -0.018 | 0.0025 | -1 | 1.00 |

f_1_ and f_2_ are the allele frequencies for variants 1 and 2, respectively.

β_1_ and β_2_ correspond to effects on eosinophil counts for variants 1 and 2, adjusted on each other.

Sign of correlation is with respect to the alleles of the variants.

LD calculations are based on 150,656 imputed Icelandic individuals that have phased genotypes.
