# Supplementary material for: A rare IL33 loss-of-function mutation reduces blood eosinophil counts and protects from asthma
Source: PLoS Genet. 2017 Mar 8;13(3):e1006659. doi: 10.1371/journal.pgen.1006659 (PMC5362243; doi:10.1371/journal.pgen.1006659)
Supplement: S2 Table — (DOCX) [file pgen.1006659.s008.docx]

**Table S2. Correlations (r^2^) between reported variants at *IL33* and the three significant eosinophil counts variants.**

|  |  |  | **rs2095044 (MAF=24.5%)** |  | **rs146597587 (MAF=0.65%)**  **splice acceptor variant** |  | **rs10758750 (MAF=27.7%)** |
| --- | --- | --- | --- | --- | --- | --- | --- |
| **Marker** | **MAF [%]** |  | **r^2^** |  | **r^2^** |  | **r^2^** |
| rs343496 | 17.4 |  | 0.017 |  | 0.0014 |  | 0.012 |
| rs7032572 | 14.3 |  | 0.46 |  | 0.0011 |  | 0.053 |
| rs72699186 | 14.1 |  | 0.47 |  | 0.0011 |  | 0.052 |
| rs1342326 | 14.1 |  | 0.47 |  | 0.0011 |  | 0.052 |
| rs2381416 | 25.8 |  | 0.94 |  | 0.0022 |  | 0.0080 |
| rs928413 | 25.4 |  | 0.87 |  | 0.0022 |  | 0.014 |
