# Supplementary material for: A rare IL33 loss-of-function mutation reduces blood eosinophil counts and protects from asthma
Source: PLoS Genet. 2017 Mar 8;13(3):e1006659. doi: 10.1371/journal.pgen.1006659 (PMC5362243; doi:10.1371/journal.pgen.1006659)
Supplement: S3 Table — (DOCX) [file pgen.1006659.s009.docx]

**Table S3. Association results in Iceland for variants at the *IL33* and *IL1RL1* loci reported to associate with asthma, allergy or eosinophil counts in Iceland.**

|  |  |  |  |  |  |  |  | **GWAS catalog information** | | | **Eosinophil counts** | |  |  | **Asthma** | |
| --- | --- | --- | --- | --- | --- | --- | --- | --- | --- | --- | --- | --- | --- | --- | --- | --- |
| **Marker** | **Chrom.** | **Pos. (hg38)** | **A1** | **A2** | **Freq. A1 [%]** | **Gene** | **Coding effect** | **Phenotype** | **reported *P*-value** | **PMID** | **β** | ***P*** |  |  | **OR** | ***P*** |
| ***IL33* locus:** |  |  |  |  |  |  |  |  |  |  |  |  |  |  |  |  |
| rs343496 | chr9 | 6,068,077 | T | A | 17.4 | - | intergenic | Asthma and hay fever | 2×10^-6^ | 24388013 | -0.002 | 0.72 |  |  | 1.00 | 0.94 |
| rs7032572 | chr9 | 6,172,380 | G | A | 14.3 | - | intergenic | Self-reported allergy | 2×10^-9^ | 23817569 | 0.052 | 2.6×10^-19^ |  |  | 1.16 | 0.00052 |
| rs72699186 | chr9 | 6,175,855 | T | A | 14.1 | - | intergenic | Asthma and hay fever | 2×10^-9^ | 24388013 | 0.053 | 8.1×10^-20^ |  |  | 1.15 | 0.00089 |
| rs1342326 | chr9 | 6,190,076 | C | A | 14.1 | - | intergenic | Asthma | 9×10^-10^ | 20860503 | 0.054 | 4.4×10^-20^ |  |  | 1.15 | 0.00089 |
| rs2381416 | chr9 | 6,193,455 | C | A | 25.8 | - | intergenic | Asthma | 2×10^-12^ | 21804549 | 0.05 | 1.7×10^-26^ |  |  | 1.11 | 0.0038 |
| rs928413 | chr9 | 6,213,387 | G | A | 25.4 | *IL33* | upstream | Asthma (childhood onset) | 9×10^-13^ | 24241537 | 0.047 | 3.4×10^-24^ |  |  | 1.12 | 0.00068 |
|  |  |  |  |  |  |  |  |  |  |  |  |  |  |  |  |  |
| ***IL1RL1 locus:*** | |  |  |  |  |  |  |  |  |  |  |  |  |  |  |  |
| rs3771180 | chr2 | 102,337,157 | T | G | 11.4 | *IL1RL1* | upstream | Asthma | 2×10^-15^ | 21804549 | -0.056 | 1.8×10^-18^ |  |  | 0.92 | 0.071 |
| rs13408661 | chr2 | 102,338,622 | A | G | 11.4 | *IL1RL1* | intronic | Asthma | 1×10^-9^ | 23028483 | -0.056 | 1.9×10^-18^ |  |  | 0.91 | 0.067 |
| rs1420101 | chr2 | 102,341,256 | T | C | 41.0 | *IL1RL1* | intronic | Eosinophil counts | 5×10^-15^ | 19198610 | 0.047 | 4.7×10^-29^ |  |  | 1.06 | 0.058 |
| rs3771175 | chr2 | 102,343,750 | A | T | 11.2 | *IL1RL1* | 3' UTR | Allergic sensitization | 5×10^-11^ | 23817571 | -0.056 | 4.9×10^-18^ |  |  | 0.92 | 0.079 |
| rs10197862 | chr2 | 102,350,089 | G | A | 11.3 | *IL1RL1* | downstream | Asthma and hay fever | 4×10^-11^ | 24388013 | -0.056 | 2.7×10^-18^ |  |  | 0.92 | 0.081 |
| rs9807989 | chr2 | 102,354,740 | C | T | 39.0 | *IL18R1* | upstream | Asthma | 6×10^-8^ | 22561531 | -0.038 | 6.6×10^-20^ |  |  | 0.94 | 0.032 |
| rs3771166 | chr2 | 102,369,762 | A | G | 39.0 | *IL18R1* | intronic | Asthma | 3×10^-9^ | 20860503 | -0.038 | 5.0×10^-20^ |  |  | 0.93 | 0.027 |

All the variants displayed in the table were well imputed (imputation information=1.00).
