# Supplementary material for: A rare IL33 loss-of-function mutation reduces blood eosinophil counts and protects from asthma
Source: PLoS Genet. 2017 Mar 8;13(3):e1006659. doi: 10.1371/journal.pgen.1006659 (PMC5362243; doi:10.1371/journal.pgen.1006659)
Supplement: S4 Table — (DOCX) [file pgen.1006659.s010.docx]

**Table S4. Variants that have r^2^>0.8 with the intergenic variant rs2095044 in an 800kb window centered on *IL33* (chr9:5.8-6.6Mb (hg38)).**

|  |  |  |  |  |  | **LD calculations with rs2095044** | |  | **Eosinophil counts** | |
| --- | --- | --- | --- | --- | --- | --- | --- | --- | --- | --- |
| **Marker** | **chr9 pos. (hg38)** | **A1** | **A2** | **Freq. A1 [%]** | ***IL33* context** | **r^2^** | **D'** |  | **β^a^** | ***P*** |
| rs2095044^b^ | 6,192,796 | T | C | 24.5 | intergenic | 1.00 | 1.00 |  | 0.051 | 1.1×10^-26^ |
| rs2381416^c^ | 6,193,455 | C | A | 25.8 | intergenic | 0.94 | 1.00 |  | 0.050 | 1.7×10^-26^ |
| rs1888909 | 6,197,392 | T | C | 24.5 | intergenic | 1.00 | 1.00 |  | 0.050 | 1.6×10^-26^ |
| rs992969 | 6,209,697 | A | G | 24.5 | intergenic | 0.97 | 0.99 |  | 0.050 | 1.0×10^-25^ |
| rs3939286 | 6,210,099 | T | C | 24.5 | intergenic | 0.97 | 0.99 |  | 0.050 | 1.1×10^-25^ |
| rs137882320^d^ | 6,211,452 | C | !C | 73.6 | upstream | 0.88 | 0.99 |  | -0.047 | 4.0×10^-24^ |
| rs928412 | 6,213,148 | A | G | 26.0 | upstream | 0.90 | 0.99 |  | 0.048 | 7.3×10^-25^ |
| rs928413 | 6,213,387 | G | A | 25.4 | upstream | 0.87 | 0.95 |  | 0.047 | 3.4×10^-24^ |
| rs7848215 | 6,213,468 | T | C | 25.1 | upstream | 0.88 | 0.95 |  | 0.048 | 8.6×10^-25^ |

Association with eosinophil counts in Iceland is shown (N=103,104).

All variants have imputation information of 1.00.

^a^ β: Effect in SD with respect to the allele A1.

^b^ The index variant rs2095044 is included in the table.

^c^ rs2381416 is a previously reported variant at the locus (see **Table S3**).

^d^ rs137882320 is a multi-allelic variant in Iceland with four alleles and the association is done for the allele shown in column A1 against all other alleles of the marker, represented by '!C' in column A2. Besides the allele presented in the table and the reference allele CTGTTT, the other two alleles with respect to the reference allele are A1=CTGTTTTGTTT (freq. A1=2.45%, info=1.00; β=0.047, *P*=0.00040) and A1=CTGTTTTGTTTTGTTT (freq. A1=0.03%, info=0.97; β=0.17, *P*=0.17).
