# Supplementary material for: A rare IL33 loss-of-function mutation reduces blood eosinophil counts and protects from asthma
Source: PLoS Genet. 2017 Mar 8;13(3):e1006659. doi: 10.1371/journal.pgen.1006659 (PMC5362243; doi:10.1371/journal.pgen.1006659)
Supplement: S5 Table — (DOCX) [file pgen.1006659.s011.docx]

**Table S5. Loss-of-function variants in *IL33* reported in the Exome Aggregation Consortium (ExAC) dataset.**

|  |  |  |  |  |  |  | **All samples** | |  |  | **Europeans (non-Finnish)** | | | |
| --- | --- | --- | --- | --- | --- | --- | --- | --- | --- | --- | --- | --- | --- | --- |
| **RSID** | **chr9 pos. (hg19)** | **REF** | **ALT** | **HGVSp^a^** | **Consequence^b^** | **Annotation^c^** | **AC^d^** | **AN^e^** | **N_homoz_^f^** | **AF^g^** | **AC^d^** | **AN^e^** | **N_homoz_^f^** | **AF^g^** |
| rs146597587 | 6,255,967 | G | C |  | c.613-1G>C | splice acceptor | 273 | 121,102 | 2 | 0.0023 | 230 | 66,632 | 1 | 0.00345 |
| rs141509242 | 6,250,600 | G | T |  | c.217+1G>T | splice donor | 31 | 120,518 | 0 | 2.6E-04 | 29 | 66,298 | 0 | 4.4E-04 |
| . | 6,252,982 | G | T | p.Glu154Ter | c.460G>T | stop gained | 12 | 114,324 | 0 | 1.1E-04 | 12 | 62,508 | 0 | 1.9E-04 |
| . | 6,250,501 | GC | G | p.Met42CysfsTer4 | c.119_120delGCinsG | frameshift | 3 | 121,222 | 0 | 2.5E-05 | 3 | 66,660 | 0 | 4.5E-05 |
| rs145735086 | 6,253,575 | G | T | p.Glu165Ter | c.493G>T | stop gained | 3 | 121,132 | 0 | 2.5E-05 | 0 | 66,612 | 0 | 0 |
| . | 6,256,090 | TC | T | p.His246IlefsTer4 | c.736delC | frameshift | 3 | 121,194 | 0 | 2.5E-05 | 3 | 66,680 | 0 | 4.5E-05 |
| . | 6,250,548 | GA | G | p.Glu56GlyfsTer15 | c.167delA | frameshift | 2 | 121,156 | 0 | 1.7E-05 | 2 | 66,616 | 0 | 3.0E-05 |
| . | 6,253,584 | C | CA | p.His168GlnfsTer5 | c.502_503insA | frameshift | 2 | 121,114 | 0 | 1.7E-05 | 0 | 66,602 | 0 | 0 |
| . | 6,241,744 | G | A | p.Trp17Ter | c.50G>A | stop gained | 1 | 112,944 | 0 | 8.9E-06 | 1 | 61,756 | 0 | 1.6E-05 |
| . | 6,241,772 | T | A | p.Cys26Ter | c.78T>A | stop gained | 1 | 110,810 | 0 | 9.0E-06 | 1 | 60,682 | 0 | 1.6E-05 |
| . | 6,250,473 | G | C |  | c.92-1G>C | splice acceptor | 1 | 121,068 | 0 | 8.3E-06 | 0 | 66,592 | 0 | 0 |
| . | 6,251,242 | C | CACTTCATG | p.Ser111PhefsTer19 | c.320_321insACTTCATG | frameshift | 1 | 121,370 | 0 | 8.2E-06 | 0 | 66,732 | 0 | 0 |
| . | 6,252,865 | G | A |  | c.344-1G>A | splice acceptor | 1 | 121,116 | 0 | 8.3E-06 | 1 | 66,620 | 0 | 1.5E-05 |
| . | 6,252,918 | ATC | A | p.Ser133HisfsTer8 | c.396_398delATCinsA | frameshift | 1 | 120,996 | 0 | 8.3E-06 | 0 | 66,548 | 0 | 0 |
| . | 6,253,551 | G | C |  | c.470-1G>C | splice acceptor | 1 | 120,900 | 0 | 8.3E-06 | 1 | 66,528 | 0 | 1.5E-05 |
| . | 6,253,574 | T | G | p.Tyr164Ter | c.492T>G | stop gained | 1 | 121,138 | 0 | 8.3E-06 | 0 | 66,616 | 0 | 0 |
| . | 6,253,591 | C | A | p.Ser170Ter | c.509C>A | stop gained | 1 | 121,088 | 0 | 8.3E-06 | 1 | 66,594 | 0 | 1.5E-05 |
| . | 6,254,461 | G | A |  | c.521-1G>A | splice acceptor | 1 | 111,120 | 0 | 9.0E-06 | 1 | 61,542 | 0 | 1.6E-05 |
| . | 6,254,543 | ACT | A | p.Ser202CysfsTer5 | c.603_604delCT | frameshift | 1 | 116,374 | 0 | 8.6E-06 | 0 | 64,116 | 0 | 0 |
| . | 6,256,063 | TG | T | p.Gly237GlufsTer4 | c.709delG | frameshift | 1 | 121,238 | 0 | 8.2E-06 | 0 | 66,698 | 0 | 0 |

**^a^**HGVS protein change annotation. **^b^**Transcript consequence according to ENST00000381434. **^c^**Variant Effect Predictor (VEP) annotation; worst across all transcripts of *IL33* (ENSG00000100399). **^d^**Allele count: alternate allele count in genotypes (genotype quality >= 20 and depth >= 10). **^e^**Allele number: total number of called genotypes (genotype quality >= 20 and depth >= 10). **^f^**Number of homozygotes for the alternative allele. **^g^**Alternative allele frequency. Table retrieved from the ExAC browser, accessed November 24th, 2015 (URL: http://exac.broadinstitute.org/gene/ENSG00000137033).
