# Supplementary material for: A rare IL33 loss-of-function mutation reduces blood eosinophil counts and protects from asthma
Source: PLoS Genet. 2017 Mar 8;13(3):e1006659. doi: 10.1371/journal.pgen.1006659 (PMC5362243; doi:10.1371/journal.pgen.1006659)
Supplement: S6 Table — (DOCX) [file pgen.1006659.s012.docx]

**Table S6. Predicted loss-of-function, missense and splice region variants in *IL33* that are detected, imputed and tested in Iceland.**

|  |  |  |  |  |  |  |  | **Eosinophil counts** | |
| --- | --- | --- | --- | --- | --- | --- | --- | --- | --- |
| **Marker** | **chr9 pos. (hg38)** | **A1** | **A2** | **Freq. A1 [%]** | **Info^a^** | ***IL33* context** | **HGVSp** | **β^b^** | ***P*** |
| rs750639062 | 6,250,477 | G | C | 0.037 | 1.00 | missense | NP_001186569.1:p.Ser32Cys, NP_254274.1:p.Ser32Cys | -0.117 | 0.31 |
| rs141509242 | 6,250,600 | T | G | 0.062 | 0.99 | splice donor | . | -0.029 | 0.73 |
| rs113609242^c^ | 6,254,555 | TAA | !TAA | 2.47 | 0.99 | splice region | . | 0.000 | 0.99 |
| rs776795276^c^ | 6,254,555 | T | !T | 0.68 | 0.79 | splice region | . | 0.033 | 0.23 |
| rs146597587 | 6,255,967 | C | G | 0.65 | 1.00 | splice acceptor | . | -0.214 | 2.5×10^-16^ |
| rs749588287 | 6,256,128 | C | T | 0.052 | 1.00 | missense | NP_001186569.1:p.Leu216Ser, NP_001186570.1:p.Leu132Ser, NP_254274.1:p.Leu258Ser | 0.001 | 0.99 |

Association with eosinophil counts in Iceland is shown (N=103,104).

^a^ Imputation information.

^b^ β: Effect in SD with respect to the allele A1.

^c^ rs113609242 and rs776795276 are two alleles of a multi-allelic variant in Iceland with three alleles. The association is done for the allele shown in column A1 against all other alleles of the marker, represented by '!T' and '!TAA' in column A2; the reference allele is TA (allele frequency 96.8%, info=0.95; β=-0.01, *P*=0.62).
