# Supplementary material for: A rare IL33 loss-of-function mutation reduces blood eosinophil counts and protects from asthma
Source: PLoS Genet. 2017 Mar 8;13(3):e1006659. doi: 10.1371/journal.pgen.1006659 (PMC5362243; doi:10.1371/journal.pgen.1006659)
Supplement: S7 Table — (DOCX) [file pgen.1006659.s013.docx]

**Table S7: Variants that have r^2^>0.8 with the splice acceptor variant rs146597587 in an 800kb window centered on *IL33* (chr9:5.8-6.6Mb (hg38)).**

|  |  |  |  |  |  |  | **LD calculations with rs146597587** | |  | **Eosinophil counts** | |
| --- | --- | --- | --- | --- | --- | --- | --- | --- | --- | --- | --- |
| **Marker** | **chr9 pos. (hg38)** | **A1** | **A2** | **Freq. A1 [%]** | **Gene** | **Gene context** | **r^2^** | **D'** |  | **β^a^ [SD]** | ***P*** |
| rs139499742 | 6,177,719 | A | C | 0.646 | - | intergenic | 0.994 | 1.00 |  | -0.212 | 4.7×10^-16^ |
| rs149045797 | 6,252,690 | G | T | 0.646 | *IL33* | intronic | 1.000 | 1.00 |  | -0.214 | 2.5×10^-16^ |
| rs146597587^b^ | 6,255,967 | C | G | 0.646 | *IL33* | splice acceptor | 1.000 | 1.00 |  | -0.214 | 2.5×10^-16^ |
| rs201552969 | 6,262,944 | C | G | 0.646 | *IL33* | downstream | 1.000 | 1.00 |  | -0.214 | 2.5×10^-16^ |
| rs370820588 | 6,263,444 | C | G | 0.646 | - | intergenic | 1.000 | 1.00 |  | -0.214 | 2.5×10^-16^ |
| rs143215670 | 6,264,551 | C | G | 0.646 | - | intergenic | 1.000 | 1.00 |  | -0.214 | 2.5×10^-16^ |
| rs201998351 | 6,268,581 | C | T | 0.646 | - | intergenic | 1.000 | 1.00 |  | -0.214 | 2.5×10^-16^ |
| rs145697747 | 6,288,604 | C | G | 0.646 | - | intergenic | 1.000 | 1.00 |  | -0.214 | 2.5×10^-16^ |
| rs185705622 | 6,291,948 | C | G | 0.646 | - | intergenic | 1.000 | 1.00 |  | -0.214 | 2.5×10^-16^ |
| rs186174039 | 6,292,798 | C | G | 0.646 | - | intergenic | 1.000 | 1.00 |  | -0.214 | 2.5×10^-16^ |
| rs564745363 | 6,298,298 | C | G | 0.646 | - | intergenic | 1.000 | 1.00 |  | -0.214 | 2.5×10^-16^ |
| rs150883069 | 6,377,725 | T | C | 0.644 | - | intergenic | 0.998 | 1.00 |  | -0.213 | 3.4×10^-16^ |
| rs373183506 | 6,414,150 | A | ACT | 0.638 | *UHRF2* | intronic | 0.988 | 1.00 |  | -0.215 | 2.7×10^-16^ |

Association with eosinophil counts in Iceland is shown (N=103,104).

All variants have imputation information of 1.00.

^a^ β: Effect in SD with respect to the allele A1.

^b^ The index variant rs146597587 is included in the table.
