# Supplementary material for: A rare IL33 loss-of-function mutation reduces blood eosinophil counts and protects from asthma
Source: PLoS Genet. 2017 Mar 8;13(3):e1006659. doi: 10.1371/journal.pgen.1006659 (PMC5362243; doi:10.1371/journal.pgen.1006659)
Supplement: S8 Table — (DOCX) [file pgen.1006659.s014.docx]

**Table S8. Variants that have r^2^>0.8 with the intronic variant rs10758750 in a 800kb window centered on *IL33* (chr9:5.8-6.6Mb (hg38)).**

|  |  |  |  |  |  |  | **LD calculations with rs10758750** | |  | **Eosinophil counts** | |
| --- | --- | --- | --- | --- | --- | --- | --- | --- | --- | --- | --- |
| **Marker** | **chr9 pos. [hg38]** | **A1** | **A2** | **Freq. A1 [%]** | **Gene** | **Gene context** | **r^2^** | **D'** |  | **β^a^ [SD]** | ***P*** |
| rs2210464 | 6,223,903 | A | T | 74.8 | *IL33* | intronic | 0.86 | 0.99 |  | 0.016 | 0.00080 |
| rs10815377 | 6,224,971 | C | G | 72.0 | *IL33* | intronic | 0.98 | 1.00 |  | 0.016 | 0.00063 |
| rs10815378 | 6,224,977 | T | C | 72.0 | *IL33* | intronic | 0.98 | 1.00 |  | 0.016 | 0.00063 |
| rs10435816 | 6,225,535 | A | G | 72.0 | *IL33* | intronic | 0.98 | 1.00 |  | 0.016 | 0.00062 |
| rs10815379 | 6,225,825 | C | A | 74.8 | *IL33* | intronic | 0.86 | 0.99 |  | 0.016 | 0.00054 |
| rs10815380 | 6,226,086 | C | T | 74.6 | *IL33* | intronic | 0.87 | 0.99 |  | 0.016 | 0.00049 |
| rs1418386 | 6,226,207 | C | T | 72.0 | *IL33* | intronic | 0.98 | 1.00 |  | 0.016 | 0.00062 |
| rs10815381 | 6,226,289 | A | G | 74.6 | *IL33* | intronic | 0.87 | 0.99 |  | 0.016 | 0.00051 |
| rs1418385 | 6,226,295 | C | T | 72.0 | *IL33* | intronic | 0.98 | 1.00 |  | 0.016 | 0.00062 |
| rs10975497 | 6,226,592 | C | T | 72.0 | *IL33* | intronic | 0.98 | 1.00 |  | 0.015 | 0.00092 |
| rs10975498 | 6,226,688 | T | C | 72.0 | *IL33* | intronic | 0.98 | 1.00 |  | 0.016 | 0.00062 |
| rs2210463 | 6,227,752 | A | G | 72.0 | *IL33* | intronic | 0.98 | 1.00 |  | 0.016 | 0.00067 |
| rs2026990 | 6,228,694 | A | G | 72.0 | *IL33* | intronic | 0.98 | 1.00 |  | 0.016 | 0.00065 |
| rs4740840 | 6,229,110 | A | G | 72.1 | *IL33* | intronic | 0.98 | 0.99 |  | 0.015 | 0.00077 |
| rs10975499 | 6,230,072 | G | A | 74.8 | *IL33* | intronic | 0.88 | 1.00 |  | 0.016 | 0.00059 |
| rs10758750^b^ | 6,230,513 | C | G | 72.3 | *IL33* | intronic | 1.00 | 1.00 |  | 0.016 | 0.00051 |
| rs10815383 | 6,230,670 | C | G | 72.3 | *IL33* | intronic | 1.00 | 1.00 |  | 0.016 | 0.00056 |
| rs7025417 | 6,240,084 | T | C | 74.8 | *IL33* | intronic | 0.88 | 1.00 |  | 0.017 | 0.00045 |
| rs10975512 | 6,243,819 | T | C | 74.8 | *IL33* | intronic | 0.88 | 1.00 |  | 0.017 | 0.00041 |
| rs10975542 | 6,314,293 | T | A | 74.9 | . | intergenic | 0.84 | 0.98 |  | 0.017 | 0.00041 |

Association with eosinophil counts in Iceland is shown (N=103,104).

All variants have imputation information of 1.00.

^a^ β: Effect in SD with respect to the allele A1.

^b^ The index variant rs10758750 is included in the table.
