# Supplementary material for: A rare IL33 loss-of-function mutation reduces blood eosinophil counts and protects from asthma
Source: PLoS Genet. 2017 Mar 8;13(3):e1006659. doi: 10.1371/journal.pgen.1006659 (PMC5362243; doi:10.1371/journal.pgen.1006659)
Supplement: S9 Table — (DOCX) [file pgen.1006659.s015.docx]

**Table S9. Correlations (r^2^) of reported variants at *IL1RL1* with the two variants from stepwise regression and two top coding signals.**

|  |  |  |  | ***Top variants from stepwise regression*** | | |  | ***Coding variants*** | | |
| --- | --- | --- | --- | --- | --- | --- | --- | --- | --- | --- |
|  |  |  |  | **rs13020553** |  | **rs6719123** |  | **rs10192157** |  | **rs1041973** |
|  |  |  | **MAF:** | **41.9%** |  | **14.2%** |  | **39.0%** |  | **17.70%** |
| **Marker** | **MAF [%]** | **Equivalence class^a^** |  | **r^2^** |  | **r^2^** |  | **r^2^** |  | **r^2^** |
| rs3771180 | 11.4 | I |  | 0.093 |  | 0.69 |  | 0.20 |  | 0.60 |
| rs13408661 | 11.4 | I |  | 0.093 |  | 0.69 |  | 0.20 |  | 0.60 |
| rs1420101 | 41.0 | III |  | 0.96 |  | 0.034 |  | 0.38 |  | 0.0025 |
| rs3771175 | 11.2 | I |  | 0.091 |  | 0.68 |  | 0.20 |  | 0.59 |
| rs10197862 | 11.3 | I |  | 0.092 |  | 0.67 |  | 0.20 |  | 0.58 |
| rs9807989 | 39.0 | II |  | 0.35 |  | 0.12 |  | 1.00 |  | 0.06 |
| rs3771166 | 39.0 | II |  | 0.35 |  | 0.12 |  | 1.00 |  | 0.06 |

^a^ Equivalence classes for reported variants:

I: rs3771180, rs13408661, rs3771175 and rs10197862 have r^2^>0.97, pairwise;

II: rs9807989 and rs3771166 have r^2^=1.00;

III: single variant.

The variants in equivalence class I are on the background of the variants equivalence class II (r^2^=0.20, positive correlation for minor alleles, D'=1.00, for rs3771180 and rs9807989).

The variants in equivalence class I are on the background of the major allele of rs1420101 (rs3771180 and rs1420101 have r^2^=0.089 with negative correlation for minor alleles and D'=1.00).
