# Supplementary material for: A rare IL33 loss-of-function mutation reduces blood eosinophil counts and protects from asthma
Source: PLoS Genet. 2017 Mar 8;13(3):e1006659. doi: 10.1371/journal.pgen.1006659 (PMC5362243; doi:10.1371/journal.pgen.1006659)
Supplement: S10 Table — (DOCX) [file pgen.1006659.s016.docx]

**Table S10. Conditional analysis based on eosinophil counts for top variants from stepwise regression and reported variants at the *IL1RL1* locus.**

|  |  |  |  |  |  | **Adjusting for top variants from stepwise regression** | | | | | |  | **Adjusting for GWAS variants** | | | | | | | |
| --- | --- | --- | --- | --- | --- | --- | --- | --- | --- | --- | --- | --- | --- | --- | --- | --- | --- | --- | --- | --- |
|  |  |  | **unadjusted** | |  | **rs13020553** | | **rs6719123** | | **rs13020553 and rs6719123** | |  | **rs3771180** | | **rs3771166** | | **rs1420101** | | **adjusted for three GWAS variants** | |
| **Marker** | **A1** | **Freq. A1 [%]** | **β** | ***P*** |  | **β** | ***P*** | **β** | ***P*** | **β** | ***P*** |  | **β** | ***P*** | **β** | ***P*** | **β** | ***P*** | **β** | ***P*** |
| *Top variants from stepwise regression:* | | | | |  |  |  |  |  |  |  |  |  |  |  |  |  |  |  |  |
| rs13020553 | G | 41.9 | 0.048 | 3.5×10^-31^ |  | - | - | 0.043 | 1.6×10^-24^ | - | - |  | 0.041 | 6×10^-21^ | 0.04 | 1.4×10^-14^ | 0.072 | 0.00094 | 0.068 | 0.002 |
| rs6719123 | G | 14.2 | -0.048 | 1.3×10^-16^ |  | -0.04 | 7.0×10^-10^ | - | - | - | - |  | -0.018 | 0.079 | -0.034 | 6.8×10^-8^ | -0.037 | 2.8×10^-10^ | -0.03 | 0.0048 |
|  |  |  |  |  |  |  |  |  |  |  |  |  |  |  |  |  |  |  |  |  |
| *GWAS variants:* | |  |  |  |  |  |  |  |  |  |  |  |  |  |  |  |  |  |  |  |
| rs3771180 | T | 11.4 | -0.056 | 1.8×10^-18^ |  | -0.04 | 3.7×10^-8^ | -0.039 | 0.00067 | -0.009 | 0.48 |  | - | - | -0.037 | 1.8×10^-7^ | -0.038 | 1.2×10^-8^ | - | - |
| rs3771166 | A | 39.0 | -0.038 | 5.0×10^-20^ |  | -0.02 | 0.0039 | -0.030 | 2.3×10^-11^ | -0.006 | 0.28 |  | -0.027 | 4.8×10^-9^ | - | - | -0.016 | 0.0032 | - | - |
| rs1420101 | T | 41.0 | 0.047 | 4.7×10^-29^ |  | -0.03 | 0.25 | 0.042 | 8.7×10^-23^ | -0.023 | 0.29 |  | 0.039 | 2.7×10^-19^ | 0.037 | 1.7×10^-12^ | - | - | - | - |

Association with eosinophil counts in Iceland is shown (N=103,104).
