# Supplementary material for: A rare IL33 loss-of-function mutation reduces blood eosinophil counts and protects from asthma
Source: PLoS Genet. 2017 Mar 8;13(3):e1006659. doi: 10.1371/journal.pgen.1006659 (PMC5362243; doi:10.1371/journal.pgen.1006659)
Supplement: S11 Table — (DOCX) [file pgen.1006659.s017.docx]

**Table S11: Variants that have r^2^>0.8 with the intronic variant rs13020553 in an 800kb window centered on *IL1RL1* (101.9-102.7Mb).**

|  |  |  |  |  |  |  | **LD calculations with rs13020553** | |  | **Eosinophil counts** | |
| --- | --- | --- | --- | --- | --- | --- | --- | --- | --- | --- | --- |
| **Marker** | **chr2 pos. [hg38]** | **A1** | **A2** | **Freq. A1 [%]** | **Gene** | **Gene context** | **r^2^** | **D'** |  | **β^a^ [SD]** | ***P*** |
| rs55966218 | 102,307,397 | T | TCTTTC | 53.9 | *IL1RL1* | upstream | 0.83 | 0.98 |  | -0.038 | 3.5×10^-20^ |
| rs12470864 | 102,309,902 | G | A | 58.1 | *IL1RL1* | upstream | 1.00 | 1.00 |  | -0.048 | 4.2×10^-31^ |
| rs13020553^b^ | 102,315,366 | C | G | 58.1 | *IL1RL1* | intronic | 1.00 | 1.00 |  | -0.048 | 3.5×10^-31^ |
| rs950880 | 102,316,102 | C | A | 58.1 | *IL1RL1* | intronic | 1.00 | 1.00 |  | -0.048 | 6.2×10^-31^ |
| rs13001325 | 102,322,576 | C | T | 58.1 | *IL1RL1* | intronic | 1.00 | 1.00 |  | -0.048 | 4.5×10^-31^ |
| rs1420104 | 102,332,010 | G | A | 58.1 | *IL1RL1* | intronic | 1.00 | 1.00 |  | -0.048 | 4.4×10^-31^ |
| rs12479210 | 102,332,701 | C | T | 58.1 | *IL1RL1* | upstream | 1.00 | 1.00 |  | -0.048 | 4.4×10^-31^ |
| rs13019081 | 102,334,362 | A | C | 58.3 | *IL1RL1* | upstream | 0.99 | 1.00 |  | -0.048 | 1.2×10^-30^ |
| rs1420101 | 102,341,256 | C | T | 59.0 | *IL1RL1* | intronic | 0.96 | 1.00 |  | -0.047 | 4.7×10^-29^ |
| rs13001714 | 102,344,025 | A | G | 57.5 | *IL1RL1* | 3' UTR | 0.81 | 0.91 |  | -0.042 | 3.3×10^-24^ |
| rs12712142 | 102,344,124 | C | A | 57.5 | *IL1RL1* | 3' UTR | 0.81 | 0.91 |  | -0.042 | 3.3×10^-24^ |
| rs6543119 | 102,346,612 | A | T | 57.5 | *IL1RL1* | downstream | 0.81 | 0.91 |  | -0.042 | 3.3×10^-24^ |
| rs13017455 | 102,348,282 | C | T | 57.5 | *IL1RL1* | downstream | 0.81 | 0.91 |  | -0.042 | 3.2×10^-24^ |
| rs11123923 | 102,351,384 | C | A | 57.5 | *IL18R1* | upstream | 0.81 | 0.91 |  | -0.042 | 2.9×10^-24^ |
| rs12999364 | 102,357,669 | C | T | 57.5 | *IL18R1* | intronic | 0.81 | 0.91 |  | -0.042 | 3.7×10^-24^ |
| rs12998521 | 102,357,957 | G | T | 57.5 | *IL18R1* | intronic | 0.81 | 0.91 |  | -0.042 | 3.6×10^-24^ |
| rs35998096 | 102,358,005 | TA | T | 57.5 | *IL18R1* | intronic | 0.81 | 0.91 |  | -0.042 | 3.7×10^-24^ |
| rs12987977 | 102,358,876 | T | G | 57.5 | *IL18R1* | intronic | 0.81 | 0.91 |  | -0.042 | 3.6×10^-24^ |
| rs6710885 | 102,361,077 | A | G | 57.5 | *IL18R1* | intronic | 0.81 | 0.91 |  | -0.042 | 3.7×10^-24^ |
| rs2287037 | 102,362,568 | C | T | 57.5 | *IL18R1* | intronic | 0.81 | 0.91 |  | -0.042 | 3.7×10^-24^ |
| rs4851569 | 102,366,787 | C | A | 57.5 | *IL18R1* | intronic | 0.81 | 0.91 |  | -0.042 | 3.7×10^-24^ |
| rs1420098 | 102,367,819 | T | C | 57.5 | *IL18R1* | splice region | 0.81 | 0.91 |  | -0.042 | 3.8×10^-24^ |
| rs1882348 | 102,368,211 | T | A | 57.5 | *IL18R1* | intronic | 0.81 | 0.91 |  | -0.042 | 3.8×10^-24^ |

Association with eosinophil counts in Iceland is shown (N=103,104). All variants in the table have imputation information of 1.00 except rs55966218 that has imputation information 0.97.

^a^ β: Effect in SD with respect to the allele A1.

^b^ The index variant rs13020553 is included in the table.
