# Supplementary material for: A rare IL33 loss-of-function mutation reduces blood eosinophil counts and protects from asthma
Source: PLoS Genet. 2017 Mar 8;13(3):e1006659. doi: 10.1371/journal.pgen.1006659 (PMC5362243; doi:10.1371/journal.pgen.1006659)
Supplement: S12 Table — (DOCX) [file pgen.1006659.s018.docx]

**Table S12: Variants that have r^2^>0.8 with the intergenic variant rs6719123** **in an 800kb window centered on *IL1RL1* (101.9-102.7Mb).**

|  |  |  |  |  |  |  | **LD calculations with rs6719123** | |  | **Eosinophil counts** | |
| --- | --- | --- | --- | --- | --- | --- | --- | --- | --- | --- | --- |
| **Marker** | **chr2 pos. [hg38]** | **A1** | **A2** | **Freq. A1 [%]** | **Gene** | **Gene context** | **r^2^** | **D'** |  | **β^a^ [SD]** | ***P*** |
| rs10178214 | 102,242,461 | G | T | 85.8 | *IL1RL2* | downstream | 1.00 | 1.00 |  | 0.048 | 1.4×10^-16^ |
| rs13027294 | 102,243,614 | G | C | 85.8 | *IL1RL2* | downstream | 1.00 | 1.00 |  | 0.048 | 1.4×10^-16^ |
| rs11677452 | 102,248,776 | A | T | 85.8 | . | intergenic | 1.00 | 1.00 |  | 0.048 | 1.4×10^-16^ |
| rs9646944 | 102,249,415 | G | C | 85.8 | . | intergenic | 1.00 | 1.00 |  | 0.048 | 1.4×10^-16^ |
| rs6719123^b^ | 102,259,080 | C | G | 85.8 | . | intergenic | 1.00 | 1.00 |  | 0.048 | 1.3×10^-16^ |

Association with eosinophil counts in Iceland is shown (N=103,104).

All variants in the table have imputation information of 1.00.

^a^ β: Effect in SD with respect to the allele A1.

^b^ The index variant rs6719123 is included in the table.
