# Supplementary material for: A rare IL33 loss-of-function mutation reduces blood eosinophil counts and protects from asthma
Source: PLoS Genet. 2017 Mar 8;13(3):e1006659. doi: 10.1371/journal.pgen.1006659 (PMC5362243; doi:10.1371/journal.pgen.1006659)
Supplement: S13 Table — (DOCX) [file pgen.1006659.s019.docx]

| **Variant 1** | | |  | **Variant 2** | | |  |  |  |
| --- | --- | --- | --- | --- | --- | --- | --- | --- | --- |
| **Marker** | **MAF [%]** | **Comment** |  | **Marker** | **MAF [%]** | **Comment** | **r^2^** | **sign** | **D'** |
| rs13020553 | 41.9 | 1^st^ variant in stepwise regression |  | rs6719123 | 14.2 | 2^nd^ variant in stepwise regression | 0.037 | -1 | 0.55 |
| rs13020553 | 41.9 | 1^st^ variant in stepwise regression |  | rs10192157 | 39.0 | missense (p.Thr549Ile) | 0.35 | -1 | 0.87 |
| rs13020553 | 41.9 | 1^st^ variant in stepwise regression |  | rs1041973 | 17.7 | missense (p.Ala78Glu) | 0.0034 | -1 | 0.15 |
| rs6719123 | 14.2 | 2^nd^ variant in stepwise regression |  | rs10192157 | 39.0 | missense (p.Thr549Ile) | 0.12 | 1 | 0.69 |
| rs6719123 | 14.2 | 2^nd^ variant in stepwise regression |  | rs1041973 | 17.7 | missense (p.Ala78Glu) | 0.68 | 1 | 0.94 |
| rs10192157 | 39.0 | missense (p.Thr549Ile) |  | rs1041973 | 17.7 | missense (p.Ala78Glu) | 0.058 | 1 | 0.41 |

**Table S13. Correlations (r^2^) for the two variants from stepwise regression and two top coding signals at *IL1RL1*.**
