# Supplementary material for: A rare IL33 loss-of-function mutation reduces blood eosinophil counts and protects from asthma
Source: PLoS Genet. 2017 Mar 8;13(3):e1006659. doi: 10.1371/journal.pgen.1006659 (PMC5362243; doi:10.1371/journal.pgen.1006659)
Supplement: S14 Table — (DOCX) [file pgen.1006659.s020.docx]

**Table S14. Conditional analysis based on eosinophil counts for top variants from stepwise regression and top coding variants at the *IL1RL1* locus.**

|  |  |  |  |  | |  | **Adjusting for top variants from stepwise regression** | | | | | |  | **Adjusting for top *IL1RL1* coding variants** | | | | | |
| --- | --- | --- | --- | --- | --- | --- | --- | --- | --- | --- | --- | --- | --- | --- | --- | --- | --- | --- | --- |
|  |  |  |  | **unadjusted** | |  | **rs13020553** | | **rs6719123** | | **rs13020553 and rs6719123** | |  | **rs10192157** | | **rs1041973** | | **rs10192157 and rs1041973** | |
| **Marker** | **A1** | **Freq. A1 [%]** | ***IL1RL1* context** | **β** | ***P*** |  | **β** | ***P*** | **β** | ***P*** | **β** | ***P*** |  | **β** | ***P*** | **β** | ***P*** | **β** | ***P*** |
| *Top variants from stepwise regression:* | | | | |  |  |  |  |  |  |  |  |  |  |  |  |  |  |  |
| rs13020553 | G | 41.9 | intronic | 0.048 | 3.5×10^-31^ |  | - | - | 0.043 | 1.6×10^-24^ | - | - |  | 0.039 | 1.6×10^-14^ | 0.047 | 1.6×10^-29^ | 0.042 | 4.2×10^-16^ |
| rs6719123 | G | 14.2 | intergenic | -0.048 | 1.3×10^-16^ |  | -0.037 | 7.0×10^-10^ | - | - | - | - |  | -0.034 | 7.1×10^-8^ | -0.062 | 1.9×10^-9^ | -0.043 | 7.3×10^-5^ |
|  |  |  |  |  |  |  |  |  |  |  |  |  |  |  |  |  |  |  |  |
| *Coding variants:* | | | | |  |  |  |  |  |  |  |  |  |  |  |  |  |  |  |
| rs10192157^a^ | T | 39.0 | missense | -0.038 | 4.2×10^-20^ |  | -0.015 | 0.0036 | -0.030 | 2.0×10^-11^ | -0.006 | 0.27 |  | - | - | -0.034 | 1.4×10^-15^ | - | - |
| rs1041973^b^ | A | 17.7 | missense | -0.032 | 3.5×10^-9^ |  | -0.028 | 1.7×10^-7^ | 0.015 | 0.11 | -0.002 | 0.84 |  | -0.021 | 1.5×10^-4^ | - | - | - | - |

The effect β (in SD) is with respect to allele A1. The two coding variants in the table correspond to the two signals involving coding variants in *IL1RL1* that show significant association with eosinophil counts.

^a^ rs10192157 (NP_057316.3:p.Thr549Ile) is perfectly correlated with four other variants that are annotated as missense variants in *IL1RL1* (Table SXXX; r^2^=1.00, D'=1.00 for all pairs; all five minor alleles are always observed together on the same chromosome in the Icelandic data).

^b^ HGSVp notations for the missense variant rs1041973: NP_003847.2:p.Ala78Glu, NP_057316.3:p.Ala78Glu.
