# Supplementary material for: A rare IL33 loss-of-function mutation reduces blood eosinophil counts and protects from asthma
Source: PLoS Genet. 2017 Mar 8;13(3):e1006659. doi: 10.1371/journal.pgen.1006659 (PMC5362243; doi:10.1371/journal.pgen.1006659)
Supplement: S15 Table — (DOCX) [file pgen.1006659.s021.docx]

**Table S15. Predicted loss-of-function, missense and splice region variants in *IL1RL1* that are detected, imputed and tested in Iceland.**

|  |  |  |  |  |  |  |  | **Eosinophil counts** | |
| --- | --- | --- | --- | --- | --- | --- | --- | --- | --- |
| **Marker** | **chr2 pos. (hg38)** | **A1** | **A2** | **Freq. A1 [%]** | **Info^a^** | ***IL1RL1* context** | **HGVSp** | **β^b^** | ***P*** |
| rs10192157^c^ | 102,351,896 | T | C | 39.0 | 1.00 | missense | NP_057316.3:p.Thr549Ile | -0.038 | 4.2×10^-20^ |
| rs10192036^c^ | 102,351,751 | A | C | 39.0 | 1.00 | missense | NP_057316.3:p.Gln501Lys | -0.038 | 4.2×10^-20^ |
| rs10204137^c^ | 102,351,752 | G | A | 39.0 | 1.00 | missense | NP_057316.3:p.Gln501Arg | -0.038 | 4.3×10^-20^ |
| rs4988956^c^ | 102,351,547 | A | G | 39.0 | 1.00 | missense | NP_057316.3:p.Ala433Thr | -0.038 | 4.4×10^-20^ |
| rs10206753^c^ | 102,351,902 | C | T | 39.0 | 1.00 | missense | NP_057316.3:p.Leu551Ser | -0.038 | 4.4×10^-20^ |
| rs1041973 | 102,339,008 | A | C | 17.7 | 1.00 | missense | NP_003847.2:p.Ala78Glu, NP_057316.3:p.Ala78Glu | -0.032 | 3.5×10^-9^ |
| rs13029918 | 102,340,831 | G | A | 2.33 | 1.00 | splice region | . | 0.015 | 0.26 |
| rs750166247 | 102,351,874 | T | C | 0.28 | 0.99 | missense | NP_057316.3:p.Pro542Ser | 0.038 | 0.32 |
| rs774991971 | 102,338,937 | C | CAAAAGTATTCCCACTCAGG | 0.04 | 0.90 | frameshift | NP_003847.2:p.Ser56GlufsTer11, NP_057316.3:p.Ser56GlufsTer11 | 0.098 | 0.36 |
| rs112595294 | 102,347,959 | G | A | 0.75 | 0.99 | missense | NP_057316.3:p.Ile329Val | 0.019 | 0.42 |
| rs111970215 | 102,342,258 | A | G | 0.75 | 0.99 | missense | NP_001269337.1:p.Ala99Thr, NP_003847.2:p.Ala216Thr, NP_057316.3:p.Ala216Thr | 0.019 | 0.42 |
| rs138992262 | 102,351,771 | T | G | 0.15 | 0.98 | missense | NP_057316.3:p.Gln507His | -0.019 | 0.73 |
| rs138892317 | 102,349,148 | A | G | 0.37 | 0.99 | missense | NP_057316.3:p.Arg396His | 0.010 | 0.77 |
| rs200831341 | 102,340,805 | T | C | 0.02 | 0.94 | missense | NP_001269337.1:p.Ala79Val, NP_003847.2:p.Ala196Val, NP_057316.3:p.Ala196Val | -0.032 | 0.83 |
| rs34210856 | 102,339,014 | T | C | 0.01 | 1.00 | missense | NP_003847.2:p.Ala80Val, NP_057316.3:p.Ala80Val | 0.014 | 0.95 |
| rs34225180 | 102,340,744 | A | G | 0.01 | 1.00 | missense | NP_001269337.1:p.Ala59Thr, NP_003847.2:p.Ala176Thr, NP_057316.3:p.Ala176Thr | 0.014 | 0.95 |

^a^ Imputation information.

^b^ β: Effect in SD with respect to the allele A1 (based on marginal association; i.e. no adjustment for other variants in the region).

^c^ There are five perfectly correlated variants in *IL1RL1* (r^2^=1.00, D'=1.00 for all pairs); all five minor alleles are always observed together on the same chromosome in the Icelandic data.
