# Supplementary material for: A rare IL33 loss-of-function mutation reduces blood eosinophil counts and protects from asthma
Source: PLoS Genet. 2017 Mar 8;13(3):e1006659. doi: 10.1371/journal.pgen.1006659 (PMC5362243; doi:10.1371/journal.pgen.1006659)
Supplement: S16 Table — (DOCX) [file pgen.1006659.s022.docx]

**Table S16: Fertility and longevity data for the nine imputed homozygotes for the splice acceptor variant rs146597587 in *IL33* found in Iceland.**

| **Gender** | **Number of offsprings^a^** | **Age reached^b^** |
| --- | --- | --- |
| female | 5 | 91^c^ |
| male | 5 | 89 |
| female | 6 | 83^c^ |
| male | 7 | 85 |
| female | 3 | 78 |
| male | 2 | 53 |
| male | 3 | 47 |
| male | 0 | 28 |
| female | 0 | 21 |

^a^ Number of offsprings the individual as of 2015.

^b^ Age in the year 2015 or age at death if deceased.

^c^ These two individuals are deceased – all other individuals in the table were still alive in 2015.
