# Supplementary material for: A rare IL33 loss-of-function mutation reduces blood eosinophil counts and protects from asthma
Source: PLoS Genet. 2017 Mar 8;13(3):e1006659. doi: 10.1371/journal.pgen.1006659 (PMC5362243; doi:10.1371/journal.pgen.1006659)
Supplement: S17 Table — (DOCX) [file pgen.1006659.s023.docx]

**S17 Table. Association of the common IL-33 variant rs2381416 with pediatric asthma of increasing severity.**

| **# of hospitalizations** |  | **Severity_group** | **BETA** | **SE** | **P** | **OR** | **95_L** | **95_U** | **N_total** |
| --- | --- | --- | --- | --- | --- | --- | --- | --- | --- |
| two | Lowest | s1 | 0.239 | 0.107 | 0.025 | 1.27 | 1.02 | 1.56 | 1171 |
| three |  | s2 | 0.178 | 0.114 | 0.12 | 1.19 | 0.95 | 1.49 | 1132 |
| four to five |  | s3 | 0.349 | 0.106 | 0.00109 | 1.41 | 1.14 | 1.74 | 1186 |
| six and more | Highest | s4 | 0.528 | 0.095 | 3.35E-08 | 1.69 | 1.4 | 2.04 | 1295 |
